# Bacillus Calmette-Guérin vaccination as defense against SARS-CoV-2 (BADAS): a randomized controlled trial to protect healthcare workers in the USA by enhanced trained immune responses

**DOI:** 10.1186/s13063-023-07662-w

**Published:** 2023-10-04

**Authors:** Andrew R. DiNardo, Moshe Arditi, Ashish M. Kamat, Kent J. Koster, Santiago Carrero, Tomoki Nishiguchi, Maxim Lebedev, Aaron B. Benjamin, Pablo Avalos, Marisa Lozano, Madeleine G. Moule, Brittany McCune, Baysia Herron, Malik Ladki, Daanish Sheikh, Matthew Spears, Ivan A. Herrejon, Courtney Dodge, Sathish Kumar, Robert W. Hutchison, Theresa U. Ofili, Lynne A. Opperman, Jessica A. Bernard, Seth P. Lerner, George Udeani, Gabriel Neal, Mihai G. Netea, Jeffrey D. Cirillo

**Affiliations:** 1 Global and Immigrant Health, Baylor College of Medicine, Houston, TX 77030 USA; 2 Radboud Center for Infectious Diseases, Department of Internal Medicine, Radboud University Medical Center, Nijmegen, The Netherlands; 3 Departments of Pediatrics and Biomedical Sciences, Cedars-Sinai Medical Center, Los Angeles, CA 90048 USA; 4 Department of Urology, UT MD Anderson Cancer Center, Houston, TX 77030 USA; 5 Center for Airborne Pathogen Research and Imaging, Texas A&M School of Medicine, Bryan, TX 77807 USA; 6 Cedars-Sinai Medical Center, Regenerative Medicine Institute, Los Angeles, CA 90048 USA; 7 Institute of Immunology & Infection Research, School of Biological Sciences, University of Edinburgh, Edinburgh, UK; 8 Texas A&M School of Medicine, Bryan, TX 77807 USA; 9 College of Osteopathic Medicine, Sam Houston State University, Conroe, TX 77304 USA; 10 Department of Psychological and Brain Sciences, Texas A&M University, College Station, TX 77843 USA; 11 Texas A&M School of Medicine, Round Rock, TX 78665 USA; 12 Department of Pharmacy Practice, Texas A&M School of Pharmacy, College Station, TX 77843 USA; 13 Center for Craniofacial Research and Diagnosis, Texas A&M School of Dentistry, Dallas, TX 75246 USA; 14 Department of Psychological and Brain Sciences, Texas A&M Institute for Neuroscience, Texas A&M University, College Station, TX 77843 USA; 15 Scott Department of Urology, Baylor College of Medicine, Houston, TX 77030 USA; 16 Department of Pharmacy Practice, Texas A&M School of Pharmacy, Kingsville, TX 78363 USA; 17 Primary Care and Rural Medicine, Texas A&M School of Medicine, Bryan, TX 77807 USA; 18 Radboud University Medical Center, Nijmegen, The Netherlands

**Keywords:** COVID-19, Randomized controlled trial, BCG vaccination, Immune training, Pandemic, Healthcare workers

## Abstract

**Background:**

A large epidemic, such as that observed with SARS-CoV-2, seriously challenges available hospital capacity, and this would be augmented by infection of healthcare workers (HCW). Bacillus Calmette-Guérin (BCG) is a vaccine against tuberculosis, with protective non-specific effects against other respiratory tract infections in vitro and in vivo. Preliminary analyses suggest that regions of the world with existing BCG vaccination programs have lower incidence and mortality from COVID-19. We hypothesize that BCG vaccination can reduce SARS-CoV-2 infection and disease severity.

**Methods:**

This will be a placebo-controlled adaptive multi-center randomized controlled trial. A total of 1800 individuals considered to be at high risk, including those with comorbidities (hypertension, diabetes, obesity, reactive airway disease, smokers), racial and ethnic minorities, elderly, teachers, police, restaurant wait-staff, delivery personnel, health care workers who are defined as personnel working in a healthcare setting, at a hospital, medical center or clinic (veterinary, dental, ophthalmology), and first responders (paramedics, firefighters, or law enforcement), will be randomly assigned to two treatment groups. The treatment groups will receive intradermal administration of BCG vaccine or placebo (saline) with groups at a 1:1 ratio. Individuals will be tracked for evidence of SARS-CoV-2 infection and severity as well as obtaining whole blood to track immunological markers, and a sub-study will include cognitive function and brain imaging. The majority of individuals will be followed for 6 months, with an option to extend for another 6 months, and the cognitive sub-study duration is 2 years. We will plot Kaplan-Meier curves that will be plotted comparing groups and hazard ratios and *p*-values reported using Cox proportional hazard models.

**Discussion:**

It is expected this trial will allow evaluation of the effects of BCG vaccination at a population level in high-risk healthcare individuals through a mitigated clinical course of SARS-CoV-2 infection and inform policy making during the ongoing epidemic.

**Trial registration:**

ClinicalTrials.gov NCT04348370. Registered on April 16, 2020.

**Supplementary Information:**

The online version contains supplementary material available at 10.1186/s13063-023-07662-w.

## Introduction

### Background and rationale

On 30 December 2019, a novel enveloped RNA betacoronavirus was detected from a patient with pneumonia of unknown etiology in Wuhan, the capital city of Hubei province. The pathogen was named the severe acute respiratory syndrome coronavirus-2 (SARS-CoV-2) [1]. Healthcare workers face an elevated risk of SARS-CoV-2 infection. In Wuhan, the hospital admission of SARS-CoV-2-infected patients substantially outweighed the number of physicians, leading to unsafe care and in-hospital transmission [2]. A pandemic reflects a serious threat to hospital personnel capacity, as the number of SARS-CoV-2 infected patients that require hospital care may well exceed the capacity of hospital personnel. It is imperative to ensure the safety, health, and fitness of existing hospital personnel in order to safeguard continuous patient care. Strategies to improve the clinical course of SARS-CoV-2 infection are therefore desperately needed.

Bacillus Calmette-Guérin (BCG) was developed as a vaccine against tuberculosis, but studies have shown its ability to induce protection against other infectious diseases or, so-called, non-specific effects (NSEs) [3]. Moreover, BCG has non-specific clinical protective effects: early administration of BCG vaccination leads to reduced child mortality, mainly as a result of reduced neonatal sepsis, respiratory infections, and fever [3, 4]. NSEs of BCG are not limited to children, as a recent study in adolescents has shown a 70% decrease in the incidence of respiratory tract infections in individuals vaccinated with BCG compared to placebo [5]. In addition, a small Indonesian trial has shown that consecutive BCG vaccination for 3 months reduced the incidence of acute upper tract respiratory infections by 80% (95% CI = 22–95%) [6]. The non-specific beneficial effects of BCG are not restricted to infections, as BCG has also been used in patients with bladder cancer to induce an improved reaction of the immune system, which prevents tumor recurrence and progression [7]. It has been recently demonstrated that the non-specific beneficial effects of BCG vaccination are due to epigenetic and metabolic reprogramming of innate immune cells such as myeloid cells and NK cells, leading to increased antimicrobial activity, a process termed “trained immunity” [8]. BCG has been shown to protect not only bacterial and fungal infections, but against viral infections such as influenza as well [9]. Furthermore, among humans receiving the yellow fever vaccine virus, those who had received BCG had less viremia and improved anti-viral responses compared to placebo-treated subjects [10]. Based on the capacity of BCG to (i) reduce the incidence of respiratory tract infections in children and adults; (ii) exert antiviral effects in experimental models; and (iii) reduce viremia in an experimental human model of viral infection, we hypothesize that BCG vaccination may induce (partial) protection against susceptibility to and/or severity of SARS-CoV-2 infection.

This study will evaluate the efficacy of BCG to prevent and improve the clinical course of SARS-CoV-2 infection. BCG vaccine in immunocompetent adult people is considered safe, even in latently infected adults with prior infant BCG vaccination [11]. In a randomized controlled trial that compared revaccination with BCG versus placebo, no serious adverse events were observed in the BCG arm [5]. Calculating a crude case fatality rate (CFR) by dividing deaths by cases, countries with a BCG program have a CFR of 0.13%, whereas countries without a BCG program have a CFR of 0.33%, suggesting BCG could impact CFR [12, 13].

Given the immediate threat of the SARS-CoV-2 pandemic, this trial has been designed as a pragmatic study with a highly feasible primary endpoint, which can be continuously (e.g., symptoms continuously and serology every other week) measured. This allows for the most rapid identification of a beneficial outcome that would allow healthcare workers and other high-risk individuals to benefit from the BCG vaccination intervention, if it is effective, at the earliest possible time during the pandemic.

### Objectives

#### Primary objective

The primary objective is to measure the efficacy of BCG vaccination among healthcare workers and high-risk individuals in preventing infection with SARS-CoV-2 in the USA.

#### Secondary objective

The secondary objective is to measure the efficacy of BCG vaccination among healthcare workers and high-risk individuals in mitigating the severity of COVID-19 disease in the USA.

### Trial design

The trial design is a placebo-controlled multi-center adaptive randomized controlled trial. As shown in Fig. 2 of Supplement 1, the sample size will be adapted based on the interim results of the primary endpoint.

## Methods: participants, interventions, and outcomes

### Study setting

Sites for the study include Texas A&M University Health Science Center and affiliated hospitals and clinics in College Station, Bryan, Round Rock, and Dallas, TX; Baylor College of Medicine and affiliated hospitals in Houston, TX; UT MD Anderson Cancer Center in Houston, TX; and Cedars-Sinai Medical Center in Los Angeles, CA.

### Eligibility criteria

In order to be eligible to participate in this study, a subject must meet the following criteria: adult (≥18 years); high-risk individual including healthcare workers, personnel working in a healthcare setting, at a hospital, medical center, or clinic (veterinary, dental, ophthalmology), or first responders (paramedics, firefighters, or law enforcement); individuals at high risk for severe disease including elderly and those with comorbidities including obesity (BMI > 25), elderly (age > 65 years), hypertension, diabetes, reactive airway disease, and smokers; and individuals at increased risk of infection because of decreased ability to limit exposure including racial and ethnic minorities, teachers, police, restaurant wait-staff, and delivery personnel.

A potential subject who meets any of the following criteria will be excluded from participation in this study: known allergy to (components of) the BCG vaccine or serious adverse events to prior BCG administration; known active or latent *Mycobacterium tuberculosis* or with another mycobacterial species or a history with or a suspicion of *M. tuberculosis* infection; fever (>38 C) within the past 24 h; pregnancy or planning pregnancy within 30 days of study enrollment; breastfeeding; suspicion of active viral or bacterial infection; any immunocompromised subjects: (a) subjects with known infection by the human immunodeficiency virus (HIV-1), (b) subjects with known neutropenia with less than 1500 neutrophils/mm^3^, (c) subjects with solid organ transplantation, (d) subjects with bone marrow transplantation; e) subjects under chemotherapy, (f) subjects with primary immunodeficiency; g) known severe lymphopenia with less than 400 lymphocytes/mm^3^; (h) treatment with any anti-cytokine therapies, (i) treatment with oral or intravenous steroids defined as daily doses of 10mg prednisone or equivalent for longer than 3 months, and (j) taking immunosuppressants; living with someone who is immunosuppressed or taking immunosuppressive drugs; previous documented infection with COVID-19; active solid or non-solid malignancy or lymphoma within the prior 2 years; direct involvement in the design or the execution of the study; not in possession and/or access to use of a smartphone, tablet, or computer; and inability to keep the vaccine site covered in the case of a draining pustule.

### Who will take informed consent

Consent will be taken via REDCap (Research Electronic Data Capture, Vanderbilt University) [14, 15].

### Additional consent provisions for collection and use of participant data and biological specimens

The medical history will be obtained from the patient medical record/clinical chart. Informed consent will be obtained to access these records. When information cannot be obtained or is not available from the patient medical record/clinical chart, it will be obtained via patient interview. Visual examination will be conducted solely to look for existing BCG vaccination scars. Symptom evaluation will be conducted via an electronic survey administered to participants every 7 days. Height, weight, HIV status, and pregnancy status will be collected as self-reported information. If unknown, a urine pregnancy test will be performed. Nasopharyngeal, oral, and/or rectal swabs will be collected for rt-PCR test for SARS-CoV-2 infection if a participant develops symptoms consistent with COVID-19. If a participant does not know their PPD/IGRA status from within the last 24 months (all healthcare providers should have this information), an IGRA can be performed to evaluate eligibility. Study participants have the option of donating blood via phlebotomy (for serological test for COVID-19 disease and PBMCs for immune correlates) or providing a fingerstick and dried blood spot (for serologic test for COVID-19). Data will be collected at four time points/periods: (1) after consent, (2) at baseline, (3) during the follow-up period, and (4) at study end. Data to be collected during screening includes medical history, visual exam results, results of rt-PCR, and serological test results. Data to be collected during baseline enrollment includes eligibility confirmation, demographic information, risk factors, randomization assignment, confirmation of BCG vaccination/placebo, and any immediate reactions to BCG vaccination/placebo. Subjects may also be asked to share a picture of just the injection site. Sharing the picture of the injection site is optional and will not be available to blinded staff members until the end of the study. Data to be collected during follow-up includes intermittent surveys about the presence of flu-like symptoms, rt-PCR test results if done, serological test results, if testing positive for COVID-19 information regarding their disease course, and disease outcome status.

## Interventions

### Explanation for the choice of comparators

In the USA, only the FDA-approved TICE® BCG (for intravesical use) BCG LIVE strain of the BCG (Merck) vaccine or a saline placebo will be administered. This will be given intradermally (0.1 mL) in the deltoid area. Placebo vaccine will be 0.1 mL 0.9% NaCl, which is the same amount and color as the intervention.

### Intervention description

Participants that are randomized in the active arm will receive the BCG vaccine, the FDA-approved TICE® BCG (for intravesical use) BCG LIVE strain in the USA.

### Criteria for discontinuing or modifying allocated interventions

Subjects can leave the study at any time for any reason if they wish to do so without any consequences. The investigator can decide to withdraw a subject from the study for urgent medical reasons. Participants who received placebo will be unblinded at the end of the study and pending a recommendation by the data safety monitoring board (DSMB), they will be offered the option of receiving the BCG intervention.

### Strategies to improve adherence to interventions

Patients will not be compensated for participating in this study. Participants will not be charged for the cost of the vaccination (or placebo). If a subject is not completing health surveys or attending follow-up visits, they will be contacted by study personnel to see whether they need help with either the surveys or the schedules. Every effort will be made to assist subjects with follow-up procedures and completing surveys.

### Relevant concomitant care permitted or prohibited during the trial

If a subject is injured, necessary facilities, emergency treatment, and professional services will be available to them, just as they are to the general community. In the event of injury resulting from this research, or negligence of local study personnel, the study team nor their institution are able to offer financial compensation nor to absorb the costs of medical treatment. Procedures and treatment for clinical care related to potential adverse events will be billed to the subject and/or their insurance or applicable third party. Subjects are not prohibited from receiving any care throughout the trial and are encouraged to continue their normal healthcare maintenance.

### Provisions for post-trial care

After the trial, the study team nor their institution are able to offer financial compensation nor to absorb the costs of medical treatment. Procedures and treatment for clinical care related to potential adverse events will be billed to the subject and/or their insurance or applicable third party.

### Outcomes

The results of this study will be disclosed unreservedly at the end of the study. Results that are important for public health will be notified to the competent authorities as soon as possible. The trial will be registered in a public trial registry before the first patient is consented. A description of this clinical trial will be available on http://www.ClinicalTrials.gov, as required by US Law. This website will not include information that can identify subjects. At most, the website will include a summary of the results. Prospective and current subjects can search this website at any time.

### Participant timeline

**Fig. 1:**
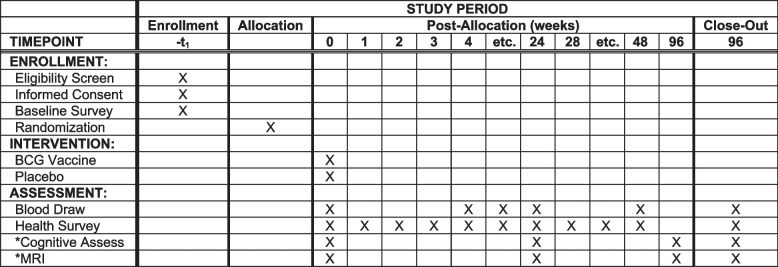
Schedule of enrollment, interventions, and assessments. *Optional assessments only conducted for subjects that select the cognitive sub-study

Subjects are screened, eligibility determined, and the informed consent is completed in REDCap in the time needed by the subject; subjects are then block randomized by study site at a 1:1 ratio vaccine:placebo group, once availability of an appropriate treatment site has been determined; the randomized subjects are scheduled for treatment, baseline screening and baseline samples are taken immediately prior to treatment, weekly health surveys are completed post-treatment, blood samples are collected every 4 weeks until week 24, qPCR testing is done upon the development of symptoms during the 24-week period, and an optional extension period of another 24 weeks is available to subjects with monthly health surveys and provision of an end of the study blood sample. Cognitive sub-study participants will also have cognitive assessments and MRI at baseline, 6 months, and 2-year time points (Fig. 1).

### Sample size

The total study will enroll ~1800 individuals. The proposed enrollment sample size is designed to provide 80% power to detect a 60% vaccine efficacy (a relative risk of 0.4 among the vaccinated). This is based on the observed threefold decline in respiratory infections in the South Africa adolescent cohort with 80% power and 0.05 type 1 error in a two-tailed test. We expect 5% of study participants to develop symptomatic COVID-19 during follow-up. We do not expect significant loss to follow-up among healthcare workers. We will plot Kaplan-Meier curves comparing COVID-19 disease between the BCG and placebo groups and report hazard ratios and *p*-values using Cox proportional hazard models.

### Recruitment

A standardized, IRB-approved email will be sent to relevant administrative centers describing the study and including a link to the screening survey. Advertisement will occur by IRB-approved postcard mailings, radio/television announcements, social media, and institutional websites. All advertising will include information or links for accessing the screening survey. A research coordinator will reach out to interested participants via phone with the help of an IRB-approved verbal script to introduce the study, confirm eligibility, and provide further instructions on how to access and sign the IRB-approved ICD via REDCap using their own electronic devices.

## Assignment of interventions: allocation

### Sequence generation

Once the eligibility is confirmed and the consent is signed by the participant and stored in REDCap, the research coordinator will randomize the participants using REDCap where access to randomization groups is blinded to all personnel other than the pharmacist preparing the treatment.

### Concealment mechanism

REDCap provides a secure data capture system that allows controlled access to treatment information, and access will be given to only the pharmacist preparing the treatment immediately prior to treatment.

### Implementation

The pharmacist will prepare the treatment and ensure coded treatments are properly allocated for subjects by trained personnel at the time of treatment.

## Assignment of interventions: blinding

### Who will be blinded

All trial participants, care providers, staff, and investigators will be blinded, except the lead pharmacist at the time of treatment and the study statistician for the preparation of DSMB reports.

### Procedure for unblinding if needed

Only the lead pharmacists will be unblinded for the implementation of treatment at the time of treatment. All participants can receive the treatment allocation at the end of the study. In case of an emergency where it is important to know the treatment received, the investigator and/or participant can reach out to the unblinded study personnel who will provide the unblinded data. Unblinding of investigators will only occur at the end of the study.

## Data collection and management

### Plans for assessment and collection of outcomes

Participants will be followed to assess whether they get infected with SARS-CoV-2. Participants will complete intermittent surveys via an electronic system every 7 days to assess the presence of any flu-like symptom, including sore throat, fever, headache, malaise, and cough. Note that this is part of routine surveillance for COVID-19 in health workers at the US site. Consent forms will ask for consent to access this survey information. Any positive response on the survey will trigger a nasopharyngeal, oral, and/or rectal swab to be collected to test for COVID-19 via rt-PCR. All participants, regardless of survey responses, will have serology for COVID-19 tested at 4-week intervals during the follow-up period (6 months). If a participant completes the follow-up period and does not test positive for a disease, their study participation is complete. If a participant does test positive for a disease, their disease status will be ascertained for up to 2 months or until an outcome is available through one of the following mechanisms: (1) an electronic survey if they are not admitted to the hospital, including questions about the number of days they are ill, daily fever, and other symptoms; or (2) if they are admitted to the hospital, ordinal outcomes for disease severity will be extracted from the hospital’s electronic medical records system. During the first week of follow-up, all participants will actively be asked about any adverse events; thereafter, participants will report unsolicited AEs through the electronic survey. Vaccine-related adverse events will be graded using the FDA guidance (https://www.fda.gov/media/73679/download) and noted using WHO-recommended Adverse event following Immunization forms (AEFI; https://vaccine-safety-training.org/classification-of-aefis.html). Participants will have the option of donating 12 mL of blood for plasma (serology) and PBMCs for secondary analysis of immune correlates and for future analysis based on COVID-19-specific IgM and IgG. If they do not donate 12 mL of blood, a fingerstick will be required for baseline COVID-19 serology. For dried blood spot (DBS), all participants will self-collect DBS samples at weeks 4, 8, 12, 16, 20, and 24. Envelopes to store the DBS are provided upon enrollment and can be mailed or dropped off at work and picked up by study coordinators. COVID-specific RNA is found in stool for ~21 days when an individual develops an infection (https://doi.org/10.1038/s41586-020-2196-x). Participants will have the option of collecting stool swabs monthly if they are asymptomatic or weekly if they develop symptoms. Nucleic acid testing will be performed in retrospect to support secondary objectives. If participants develop symptoms consistent with COVID-19, they will be PCR-tested for COVID-19. They will be given the option of donating 12 mL of blood for plasma and PBMCs 2 weeks after symptoms resolve. At week 12 (+/− 2 weeks), participants will be given the option to donate 12 mL of blood for plasma and PBMCs for secondary analysis of immune correlates and for future secondary analysis based on COVID-specific IgM and IgG. At week 24 (+/− 2 weeks), participants will be given the option to donate 12 mL of blood for plasma and PBMCs for secondary analysis of immune correlates and for future secondary analysis based on COVID-specific IgM and IgG. Except for the administration of the BCG vaccine or placebo and the abovementioned DBS and phlebotomy, participants will undergo no invasive procedures for study purposes.

### Plans to promote participant retention and complete follow-up

If a subject is not completing health surveys or attending follow-up visits, they will be contacted by study personnel to see whether they need help with either the surveys or the schedules. Every effort will be made to assist subjects with follow-up procedures and completing surveys.

### Data management

Data will be maintained in REDCap on a secure server. A subject identification code list will be used to link the data to the subject. The key to the code will be safeguarded by each site in a designated location. The electronic consent forms are completed on an ongoing basis during the study. The consent forms will be managed via REDCap.

### Confidentiality

All data submitted will be coded using numeric identifiers only. Only on-site research staff will have access to records that may identify subjects. Any paper research and clinical records will be stored on-site in a locked cabinet in a secure location. Electronic records will be accessible only by data management staff, clinical monitors, and active site personnel who have furnished the required training and credentials. Subject information will not be released without written permission, except as necessary for monitoring by the FDA.

### Plans for collection, laboratory evaluation, and storage of biological specimens for genetic or molecular analysis in this trial/future use

All specimens will be coded to the subject without personal identifiers and maintained in a repository by the investigators. The site principal investigator is responsible for maintaining accurate, complete, and up-to-date records for each subject and all specimens. The site principal investigator is also responsible for maintaining any source documentation for future use in epigenetic, immune functional studies, and other relevant analyses to provide insight into the impacts of treatment.

## Statistical methods

### Statistical methods for primary and secondary outcomes

For the primary endpoint, the development of COVID-19 infection, we will use the Cox proportional-hazards model to calculate hazard ratios for the development of COVID-19. This will be reported as the proportion of individuals receiving the intervention who are PCR-positive or seroconvert.

For the secondary endpoint, the disease severity, we will use the chi-square for significance to calculate the risk ratios for the development of severe COVID-19 disease. Disease severity will be based on the level of care required for individuals who test positive for COVID-19 as follows: non-hospital-based care; patient hospitalized but no oxygen required; hospitalized and oxygen required; patient treated in intensive care and/or on mechanical ventilation; patient died. Additional WHO criteria for severity include severe pneumonia, respiratory failure, acute respiratory distress syndrome, sepsis, and septic shock. Days absent will also be self-reported via the REDCap Research App. Immunology and epigenetic studies for innate training will be implemented, but in brief, immune cells will be stimulated with non-specific (LPS, mitogen, BCG) and COVID-19-specific antigens with immune function measured by ELISA and flow cytometry. Epigenetic studies are discussed in more detail below.

### Interim analyses

One month after the first 100 participants are enrolled, an interim analysis will be held by the independent statistician of the trial. Data will be exported independently from all sites and then data collated and merged for the interim analysis. The above-described statistical analysis will be evaluated as well as safety profile. Enrollment is expected to occur within the first 2 months of launch. Interim analysis will occur based on study enrollment, and three to six interim analyses will occur based on temporal dynamics of enrollment as requested by the DSMB. We will stop the clinical trial early if the *p*-value of any interim analysis is smaller than the pre-specified type-1 error cutoff. Each interim analysis will be reported to the DSMB.

### Methods for additional analyses (e.g., subgroup analyses)

Continuous baseline characteristics will be reported as mean and standard deviation or median and inter-quartile range, as appropriate. Categorical baseline characteristics will be reported as count and percentage. No statistical testing for baseline characteristics will be performed. Differential epigenetic or immunological parameters will be defined as greater than 2 or less than 0.5-fold difference with *p* values < 0.05.

### Methods in analysis to handle protocol non-adherence and any statistical methods to handle missing data

Data will be reported quantitatively. All analyses will be performed from the intention-to-treat principle. Missing data will be dealt with by multiple imputation using the multivariate imputation by chained equations (mice) package in R.

### Plans to give access to the full protocol, participant-level data, and statistical code

The full protocol is available as supplementary material (Supplement 1) for this publication and is publicly available. Participant-level data will be coded with numeric identifiers only. Statistical code is available by reasonable request to the principal investigator.

## Oversight and monitoring

### Composition of the coordinating center and trial steering committee

A copy of the protocol, proposed informed consent form, other written subject information, and any proposed advertising material must be submitted to the institutional review board (IRB) for the lead institution for each study site for written approval. The investigator will notify the IRB of deviations from the protocol or serious adverse events/unanticipated problems occurring in accordance with local procedures. The investigator is responsible for obtaining annual IRB approval/renewal throughout the duration of the study. The IRB will be notified of the completion or termination of this study and sent a copy of the study synopsis in accordance with applicable timelines.

### Composition of the data monitoring committee, its role, and reporting structure

Safety oversight will be provided by a DSMB. The DSMB consists of 5–7 expert members and are independent of study personnel. The DSMB will consist of members with expertise related to the trial. The DSMB meetings will be run via teleconference. If members are unable to participate, they will be replaced with another member with similar expertise. The DSMB will review study collected data and events every month including enrollment, demographics, compliance, and adverse events. The DSMB can also meet ad hoc in the event of an unanticipated significant adverse event. The DSMB independent study statistician, will have access to unblinded study data to evaluate these criteria. A final data review meeting will occur 2 to 4 months after study completion to review the cumulative safety data. Additional data can be requested by the DSMB as they deem requisite. The DSMB may share de-identified reports with participating/collaborating sites and/or institutions.

### Adverse event reporting and harms

Unexpected problems (UP) are defined according to each institutional IRB of record. The UP will be defined and reported per institutional policy. All serious adverse events will be reported to the local site PI within 24 h of knowledge of the event. Unless stated otherwise in the protocol, all significant adverse events (SAE), expected or unexpected, will be reported to the DSMB as soon as possible, but in all cases within 5 working days of knowledge of the event regardless of the attribution. Death or life-threatening events that are unexpected, possibly, probably, or definitely related to drugs must be reported within 24 h of knowledge of the event. Serious adverse events will be captured from the time of the first protocol-specific intervention, unless the protocol states otherwise, and be reported until 30 days after the last protocol-specific data collection timepoint. Serious adverse events must be followed until clinical recovery is complete and standard of care laboratory tests have returned to baseline, progression of the event has stabilized, or there has been acceptable resolution of the event. Additionally, any serious adverse events that occur after the 30-day time period or protocol-specific timeline that is related to the study treatment must be reported to the relevant IRB.

### Frequency and plans for auditing trial conduct

The Investigator will assure that the study staff cooperate with monitoring and audits. The Investigator agrees to allow auditing of all essential clinical study documents by the FDA or other appropriate regulatory authorities. Auditing visits will be scheduled with the appropriate staff at mutually agreeable times as applicable.

### Plans for communicating important protocol amendments to relevant parties (e.g., trial participants, ethical committees)

Changes must be implemented by formal protocol amendment. Protocol amendments must not be implemented without prior IRB approval. When the change(s) involve only logistic or administrative aspects of the study, the IRB only needs to be notified. The data recorded on the consent (CRF; Supplement 2) and source documents will reflect any departure from the protocol and the source documents will describe the departure and the circumstances requiring it.

### Dissemination plans

The results of this study will be disclosed unreservedly at the end of the study. Results that are important for public health will be notified to the competent authorities as soon as possible (RIVM, WMO). The trial will be registered in a public trial registry before the first patient is consented. A description of this clinical trial will be available on http://www.ClinicalTrials.gov, as required by US Law. This Web site will not include information that can identify subjects. At most, the Web site will include a summary of the results. Prospective and current subjects can search this Web site at any time.

## Discussion

The risk to and burden for the subject of BCG vaccination is estimated to be low, according to two previous trials that have been performed with BCG vaccines [6, 11, 16] The beneficial effect of BCG vaccination for the individualized participant is unknown, although the objective is to prevent severe illness to SARS-CoV-2 infection. Subsequent to the first submission of this manuscript, two studies were published reporting the absence of a protective effect of BCG on COVID-19, [17, 18], but conflicting results have been obtained [19] and based on the promising results obtained from immunological studies associated with similar clinical trials, [20, 21] further studies are needed to better understand the specific mechanisms involved. Using an adaptive design, the study aims to find a positive effect of BCG-vaccine on a population level, which could be applied quickly in participants allocated to placebo and implemented to hospitals that do not participate in the study.

### Trial status

Enrollment is completed, but data collection is ongoing, with the expectation that data collection for the cognitive sub-study will be completed in April 2023. Although it would have been optimal to publish this protocol prior to enrollment of the first subjects, logistics, acquisition of funding, organization of the trial, and completion of the primary components of data collection have prevented communication of this protocol at any earlier date.

### Supplementary Information

**Additional file 1.**

**Additional file 2.**

## Data Availability

Data will be available from the author on reasonable request and pending ethics approval (jdcirillo@tamu.edu).

## Abbreviations

AE: Adverse event
AR: Adverse reaction
BCG: Bacillus Calmette-Guérin
BCM: Baylor College of Medicine
CA: Competent authority
CFR: Case fatality rate
CRF: Consent response form
CSMC: Cedars Sinai Medical Center
DSMB: Data Safety Monitoring Board
EU: European Union
FDA: Food and Drug Administration
GCP: Good Clinical Practice
HCW: Healthcare workers
ICU: Intensive care unit
IRB: Institutional Review Board
MDACC: M.D. Anderson Cancer Center
NSE: Non-specific effects
PCR: Polymerase chain reaction
(S)AE: (Serious) adverse event
SARS-CoV-2: Severe acute respiratory syndrome coronavirus-2
RNA: Ribonucleic acid
PBMC: Peripheral blood mononuclear cell
TAMU: Texas A&M University
UP: Unanticipated problem
WHO: World Health Organization
