# Supplementary material for: Bacillus Calmette-Guérin vaccination as defense against SARS-CoV-2 (BADAS): a randomized controlled trial to protect healthcare workers in the USA by enhanced trained immune responses
Source: Trials. 2023 Oct 4;24:636. doi: 10.1186/s13063-023-07662-w (PMC10548680; doi:10.1186/s13063-023-07662-w)
Supplement: Supplementary file 1 — Additional file 1. [file 13063_2023_7662_MOESM1_ESM.docx]

**Supplementary Information**

**Supplement 1**

**BACILLUS CALMETTE-GUÉRIN VACCINATION (**TICE® BCG) **AS DEFENSE AGAINST SARS-CoV-2. A RANDOMIZED CONTROLLED TRIAL TO PROTECT HEALTH CARE WORKERS BY ENHANCED TRAINED IMMUNE RESPONSES**

**PROTOCOL TITLE:** ‘

**BACILLUS CALMETTE-GUÉRIN VACCINATION AS DEFENSE AGAINST SARS-CoV-2. A RANDOMIZED CONTROLLED TRIAL to PROTECT HEALTH CARE WORKERS BY ENHANCED TRAINED IMMUNE RESPONSES**

**Protocol ID:** BADAS

**Protocol Version:** 1.9

**Date**: 31 October 2020

**Principal Investigators:** ***Texas A&M University (TAMU):*** Jeffrey D Cirillo, PhD

***Baylor College of Medicine (BCM) and Affiliates:*** Andrew DiNardo, MD

***MD Anderson Cancer Center (MDACC):*** Ashish Kamat, MD

***Cedars Sinai Medical Center (CSMC):*** Moshe Arditi, MD

**TABLE OF CONTENTS**

1. INTRODUCTION AND RATIONALE 6

2. RESEARCH QUESTIONS 9

3. STUDY DESIGN 9

4. STUDY POPULATION 10

a. Population (base) 10

b. Inclusion criteria 11

c. Exclusion criteria 11

d. Coordination among participating sites 12

5. TREATMENT OF SUBJECTS 12

e. Investigational product/treatment 12

4. INVESTIGATIONAL PRODUCT 12

a. Name and description of investigational product(s) 12

b. Summary of findings from non-clinical studies 12

c. Summary of findings from clinical studies 12

d. Summary of known and potential risks and benefits 13

e. Route of administration and dosage 13

g. Preparation and labelling of Investigational Product (IP) 13

h. Drug accountability 14

6. STUDY PARAMETERS/ENDPOINTS 14

a. Primary study endpoint 14

b. Secondary study endpoint 14

7. STUDY PROCEDURES 14

a. Recruitment, Randomization, treatment allocation, and blinding 14

b. Baseline data collection/procedures 16

c. Follow-up procedures and data collection: 17

d. Withdrawal of individual subjects/End of Study 18

e. Replacement of individual subjects after withdrawal 18

a. Rationale: 19

b. Premature termination of the study 19

8. OPTIONAL COGNITIVE SUB-STUDY (Not available at all sites). 19

a. Population: 19

b. Additional Inclusion Criteria: 19

d. Additional Exclusion Criteria: 19

e. Study Endpoints (Additional to Main Study): 19

f. Additional Risks Beyond Main Study: 20

g. Clinical Cognitive Assessment (CCA): 20

h. Magnetic Resonance Imaging (MRI): 21

9. SAFETY REPORTING 21

a. Temporary halt for reasons of subject safety 21

b. AEs, SAEs and SUSARs 22

i. Adverse events (AEs) 22

ii. Expected mild Adverse events 22

iii. Expected rare, serious Adverse events: 22

iv. Serious adverse events (SAEs) 23

v. Suspected unexpected serious adverse reactions (SUSARs) / Unanticipated Problems (UP) 23

c. Data Safety Monitoring Board 24

d. Halting Rules 24

e. Annual safety report 24

f. Follow-up of adverse events 24

10. SAMPLE SIZE AND STATISTICAL ANALYSIS 24

a. Sample size calculation 24

b. Interim analysis 25

c. Primary study parameter(s) 25

d. Secondary study parameter(s) 25

e. Other study parameters 25

f. Synthesis 25

g. Epigenetic and Immune Corollary Studies 26

h. Flow cytometry based immune profiling 26

i. Epigenomic DNA methylation 26

j. ATAC-seq 27

k. Benefits and risks assessment, group relatedness 27

11. STRUCTURED RISK ANALYSIS 27

12. REGUALTORY REQUIREMENTS/REPORTING, MONITORING, PUBLICATION 29

a. Financial Disclosure 29

b. IRB Approval 29

c. Ethical conduct of the study 29

d. Ethics and regulatory review 29

e. Informed consent 29

f. Incentives and Research Related Injury 30

g. Changes to the Protocol and/or Informed Consent 30

h. Annual progress report 30

i. Training of site study personnel 30

j. Study Management 31

k. Data Management, Handling, Storage and Security of data and documents 31

l. Temporary halt and end of study report 31

m. Monitoring and Quality Assurance 31

n. Audits and Inspections 31

o. Confidentiality and Reporting of Results 32

p. Public disclosure and publication policy 32

13. REFERENCES 32

**LIST OF ABBREVIATIONS AND RELEVANT DEFINITIONS**

| **AE** | **Adverse Event** |
| --- | --- |
| **AR**  **BCG** | **Adverse Reaction**  **Bacillus Calmette-Guerin** |
| **BCM** | **Baylor College of Medicine** |
| **CA** | **Competent Authority** |
| **CFR** | **Case Fatality Rate** |
| **CSMC** | **Cedars Sinai Medical Center** |
| **CV** | **Curriculum Vitae** |
| **DSMB** | **Data Safety Monitoring Board** |
| **EU** | **European Union** |
| **FDA** | **Food and Drug Administration** |
| **EudraCT** | **European drug regulatory affairs Clinical Trials** |
| **GCP** | **Good Clinical Practice** |
| **HCW** | **HealthCare Workers** |
| **IB** | **Investigator’s Brochure** |
| **ICD** | **Informed Consent Document** |
| **ICF** | **Informed Consent Form** |
| **ICU** | **Intensive Care Unit** |
| **IMP** | **Investigational Medicinal Product** |
| **IRB** | **Institutional Review Board** |
| **IMPD** | **Investigational Medicinal Product Dossier** |
| **MDACC** | **M.D. Anderson Cancer Center** |
| **NSE** | **Non specific effects** |
| **PCR** | **Polymerase chain reaction** |
| **(S)AE** | **(Serious) Adverse Event** |
| **SARS-CoV-2** | **Severe Acute Respiratory Syndrome Coronavirus-2** |
| **Sponsor** | **The sponsor is the party that commissions the organization or performance of the research, for example a pharmaceutical**  **company, academic hospital, scientific organization or investigator (Sponsor-Investigator). A party that provides funding for a study but does not commission it is not regarded as the sponsor, but referred to as a subsidizing party.** |
| **RNA** | **Ribonucleic acid** |
| **PBMC** | **Peripheral blood mononuclear cell** |
| **SUSAR** | **Suspected Unexpected Serious Adverse Reaction** |
| **TAMU** | **Texas A&M University** |
| **UP** | **Unanticipated Problem** |
| **WHO** | **World Health Organization** |
|  |  |

**SUMMARY**

**Rationale:** SARS-CoV-2 spreads rapidly throughout the world. A large epidemic would seriously challenge the available hospital capacity, and this would be augmented by infection of healthcare workers (HCW). Strategies to prevent infection and disease severity of HCW are, therefore, desperately needed to safeguard continuous patient care. Bacille Calmette-Guérin (BCG) is a vaccine against tuberculosis, with protective non-specific effects against other respiratory tract infections in *in vitro* and *in vivo* studies, and reported morbidity and mortality reductions as high as 70%.[1] Furthermore, in our preliminary analysis, areas with existing BCG vaccination programs appear to have lower incidence and mortality from COVID19[2]. We hypothesize that BCG vaccination can reduce SARS infection and disease severity during the epidemic phase of SARS-CoV-2.

**Objective:** ***Primary objective:*** To measure the efficacy of BCG vaccination inhigh risk individuals (HRI) for infection with SARS-CoV2.

***Secondary objective:*** To measure the efficacy of BCG vaccination among HRI in mitigating the severity of COVID19.

**Study design:** A placebo-controlled adaptive multi-center randomized controlled trial.

**Study population:** High risk individuals (HRI) including those with comorbidities (hypertension, diabetes, obesity, reactive airway disease, smokers), racial and ethnic minorities, elderly, teachers, police, restaurant wait-staff, delivery personnel; Health Care Workers; Health Care Workers (HCW) defined as personnel working in a healthcare setting, at a hospital, medical center or clinic (veterinary, dental, ophthalmology); and first responders (paramedics, firefighters, or law enforcement).

**Intervention**: Participants will be randomized between intradermal administration of BCG vaccine or placebo in a 1:1 ratio.

**Study endpoints:** Primary endpoint: incidence of SARS-CoV2 infection following BCG vaccination compared to placebo. Secondary endpoint: COVID19-related disease severity following BCG vaccination compared to placebo.

**Nature and extent of the burden and risks associated with participation, benefit and group relatedness:** Based on previous experience and randomized controlled trials in adult and elderly individuals, the risks of BCG vaccination are considered low. The objective of this trial is to evaluate the beneficial effects of BCG vaccination at a population level of high risk health care workers through a mitigated clinical course of SARS-CoV-2 infection. The primary endpoint and the adaptive design with interim analyses facilitate maximum efficiency of the trial, so that results can inform policy making during the ongoing epidemic.

# 1. INTRODUCTION AND RATIONALE

On 30 December 2019, a novel enveloped RNA betacoronavirus was detected from a patient with pneumonia of unknown etiology in Wuhan, the capital city of Hubei province. The pathogen was named the severe acute respiratory syndrome coronavirus-2 (SARS-CoV-2).[3] Since beginning of 2020, SARS-CoV-2 spread rapidly throughout China and the rest of the world, the first detected case in the Netherlands on 27 February 2020. In the United States, there have been over 156,000 cases as of 30 March 2019.

From a cohort of patients with SARS-CoV-2 admitted to hospitals in the Wuhan region, (n=1099), a mortality rate of 1.4% was observed, with an ICU admission rate of 5% and 2.3% undergoing invasive mechanical ventilation.[3]

The estimated basic reproduction number (R0) of SARS-CoV-2 is ~2.2-2.7 and, on average, each infected person spreads the infection to an additional two persons. SARS-CoV-2 is being transmitted via droplets and fomites during close unprotected contact between an infector and infectee. According to WHO, as of 20 February 2020, 75,465 laboratory-confirmed SARS-CoV-2 cases were established. Health-care workers face an elevated risk of exposure to- and infection of- SARS-CoV-2, although in China, surprisingly, infection of health care workers could mostly be traced back to in-household transmission. Of these 75,465 laboratory-confirmed cases, 2,055 (2.7%) were reported among health care workers from 476 hospitals across China. The majority of cases (77.8%) were found in the working age (30–69 years).

In Wuhan, the hospital admission of SARS-CoV-2 infected patients substantially outweighed the number of physicians, leading to unsafe care and in-hospital transmission.[4] Consequently, in the district of Wuhan 40,000 health care workers have been deployed from other areas of China to support the response in Wuhan.

Subsequently, a SARS-CoV-2 pandemic reflects a serious threat to hospital personnel capacity, as the number of SARS-CoV-2 infected patients that require hospital care may well exceed the capacity of hospital personnel. It is imperative to ensure the safety, health and fitness of existing hospital personnel in order to safeguard continuous patient care. Strategies to improve the clinical course of SARS-CoV-2 infection are therefore desperately needed. To date, treatment for SARS-CoV-2 has been supportive, and no curative or protective treatments have been identified yet.

Bacillus Calmette-Guérin (BCG) was developed as a vaccine against tuberculosis, but many studies have shown its ability to induce potent protection against other infectious diseases: the so called non-specific effects (NSEs).[5] Moreover, BCG has non-specific clinical protective effects: early administration of BCG vaccination leads to reduced child mortality, mainly as a result of reduced neonatal sepsis, respiratory infections, and fever.[1, 5] NSEs of BCG are not limited to children, as a recent study in adolescents has shown a 70% decrease in the incidence of respiratory tract infections in individuals vaccinated with BCG compared to placebo.[6] In addition, a small Indonesian trial has shown that consecutive BCG vaccination for 3 months reduced the incidence of acute upper tract respiratory infections by 80% (95%CI=22-95%).[7] The non-specific beneficial effects of BCG are not restricted to infections, as BCG has also been used in patients with bladder cancer, to induce an improved reaction of the immune system, which prevents tumor progression and mortality.[8]

It has been recently demonstrated that the non-specific beneficial effects of BCG vaccination are due to epigenetic and metabolic reprogramming of innate immune cells such as myeloid cells and NK cells, leading to an increased antimicrobial activity, a process termed ‘trained immunity’.^12^ Upon stimulation with a pathogen, the innate immune system becomes primed and is able to react faster and more efficient to a secondary (and non-related) stimulus. In experimental studies, BCG has been shown to protect not only bacterial and fungal infections, but against viral infections such as influenza as well.[9] Furthermore, among humans receiving yellow fever vaccine virus, those who had received BCG had less viremia, and improved anti-viral responses compared to placebo treated subjects.[10] The observed effects are proposed to be due to modulation of the human innate immune system through ‘trained immunity’ and are long-lasting for at least one year

Based on the capacity of BCG to: i. reduce the incidence of respiratory tract infections in children and adults; ii. exert antiviral effects in experimental models; and iii. reduce viremia in an experimental human model of viral infection, we hypothesize that BCG vaccination may induce (partial) protection against susceptibility to and/or severity of SARS-CoV-2 infection. This study will evaluate the efficacy of BCG to prevent and improve the clinical course of SARS-CoV-2 infection.

BCG vaccine in immunocompetent adult people is considered safe, even in latently infected adults with prior infant BCG vaccination.[11] In a randomized controlled trial that compared revaccination with BCG versus placebo, no serious adverse events were observed in the BCG arm.[6]

Additionally, we have analyzed data and found that over the 15-day period from 9 March to 24 March 2020, the incidence of Covid-19 was 80 per million population, with a fatality of 0.55 per million. A total of 178 countries were in the database: current national programs of BCG vaccination exist in 131 countries; 21 countries have no current program of national BCG vaccination; and for 26 countries status is unknown. When we dichotomised the data according to those countries with and without BCG programs, the incidence of Covid-19 was 38.4 per million in countries with BCG vaccination whereas the incidence of Covid-19 was 358.4 per million in the absence of such a program. Likewise, the fatality recorded in countries with BCG programs was 4.28/million, compared to 40/million in countries without a national program. Calculating a crude case fatality rate (CFR) by dividing deaths by cases, countries with a BCG program the CFR was 0.13% and 0.33% in countries without a BCG program. Countries that have a booster injection of BCG 7 to 14 years later had no better outcomes than those with a single inoculation only. [2, 12].

**A randomized controlled trial provides the highest validity for this research question. Given the immediate threat of the SARS-CoV-2 epidemic the trial has been designed as a pragmatic study with a highly feasible primary endpoint, that can be continuously (e.g. symptoms continuously and serology every other week) measured. This allows for the most rapid identification of a beneficial outcome that would allow other HRI and HCWs (including the control population) to also benefit from the intervention if and as soon as it has been demonstrated to be effective.**

# 2. RESEARCH QUESTIONS

Primary Objective:

1. To measure the efficacy of BCG vaccination among HRI and HCW in preventing infection with SARS-CoV2 in the United States.

Secondary Objective:

1. To measure the efficacy of BCG vaccination among HRI and HCW in mitigating the severity of Covid-19 disease in the United States.

# STUDY DESIGN

A placebo-controlled multi-center adaptive randomized controlled trial, see figure 2 for the logistics of the study.

**Figure 2:** Study design of the trial


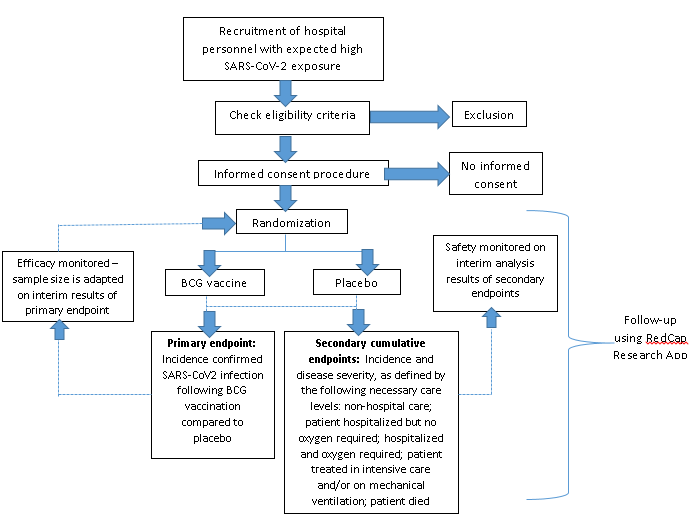


1. **Duration of Follow-Up**

The duration of follow-up for each participant depends on the interim results of the primary endpoint and the probability of obtaining a result, with a maximum of 168 days. The end of the study is defined as the last patient’s last registration in the mobile application. Study participants will collect fingerstick dried blood spot (DBS) samples for serology testing at baseline, weeks 4, 8, 12, 16, 20 and 24. If they develop symptoms, they will be qPCR tested by nasopharyngeal swab, oral swab and/ or fecal swabs.

# STUDY POPULATION

## Population (base)

BCG vaccination 2 to 4 weeks before exposure to SARS-CoV-2 would lead to an optimal immunologic response and best expected clinical effects. As this time point cannot be determined (and may have already passed), participating hospitals will be selected by the study team based on their capability to perform study-related procedures. Currently, rapid tests to evaluate participants previous exposure to COVID19 do not exist, but will emerge in the coming weeks. Blood will be obtained and banked, and serologic status will be evaluated retrospectively.

In-hospital recruitment of hospital personnel will be done in different phases. The selection is based on the likelihood of taking care for patients with SARS-CoV-2 infection and, thus, the risk of SARS-CoV-2 exposure, see figure 3 for an example of recruitment.

**Figure 3:**Recruitment of subjects for the trial:


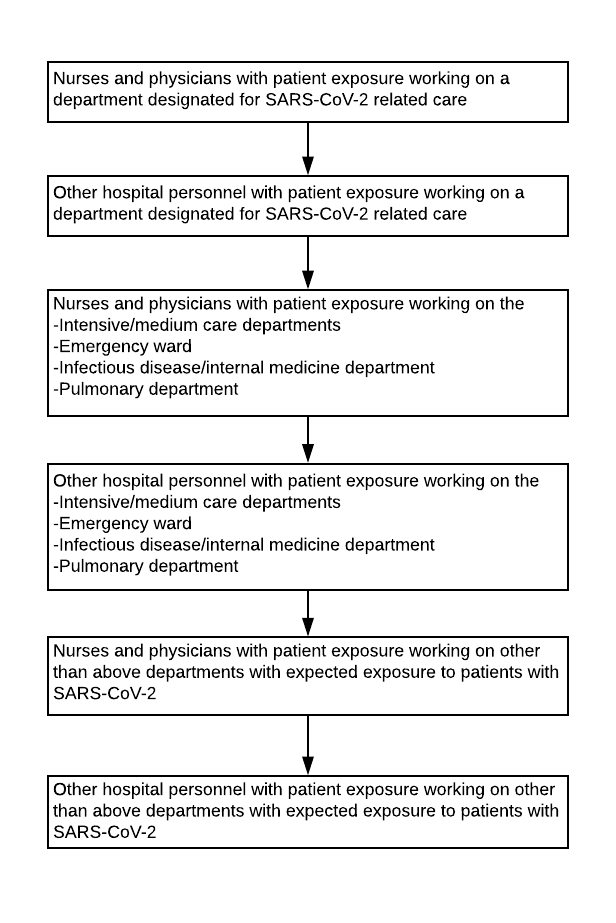


## Inclusion criteria

In order to be eligible to participate in this study, a subject must meet the following criteria:

- Adult (≥18 years)
- Male or female
- High risk individual including:
- Health Care Workers (HCW) Personnel working in a healthcare setting, at a hospital, medical center or clinic (veterinary, dental, ophthalmology), or first responders (paramedics, firefighters, or law enforcement).
  1. High risk for severe disease including elderly and those with comorbidities including obesity (BMI > 25), elderly (age > 65 years), hypertension, diabetes, reactive airway disease, smokers
  2. Individuals at increased risk of infection because of decreased ability to limit exposure including racial and ethnic minorities, teachers, police, restaurant wait-staff, delivery personnel

## Exclusion criteria

A potential subject who meets any of the following criteria will be excluded from participation in this study:

- Known allergy to (components of) the BCG vaccine or serious adverse events to prior BCG administration
- Known active or latent *Mycobacterium tuberculosis* or with another mycobacterial species. A history with- or a suspicion of *M. tuberculosis* infection.
- Fever (>38 C) within the past 24 hours
- Pregnancy or planning pregnancy within 30 days of study enrollment
- Breastfeeding
- Suspicion of active viral or bacterial infection
- Any Immunocompromised subjects. This exclusion category comprises: a) subjects with known infection by the human immunodeficiency virus (HIV-1); b) subjects with known neutropenic with less than 1500 neutrophils/mm3; c) subjects with solid organ transplantation; d) subjects with bone marrow transplantation; e) subjects under chemotherapy; f) subjects with primary immunodeficiency; g) known severe lymphopenia with less than 400 lymphocytes/mm3; h) treatment with any anti-cytokine therapies; i) treatment with oral or intravenous steroids defined as daily doses of 10mg prednisone or equivalent for longer than 3 months; j) taking immunosuppressants
- Living with someone who is immunosuppressed or taking immunosuppressive drugs
- Previous documented infection with COVID19
- Active solid or non-solid malignancy or lymphoma within the prior two years
- Direct involvement in the design or the execution of the study
- Not in possession and/or access to use of a smartphone, tablet or computer
- Inability to keep the vaccine site covered in the case of a draining pustule.

**Secondary analysis**: Disease severity will be based on the level of care required for individuals who test positive for COVID19 as follows: non-hospital-based care; patient hospitalized but no oxygen required; hospitalized and oxygen required; patient treated in intensive care and/or on mechanical ventilation; patient died. Additional WHO criteria for severity include severe pneumonia, respiratory failure, acute respiratory distress syndrome, sepsis and septic shock. Days absent will be self-reported via the CRF. Immunology and epigenetic studies for innate training be implemented as previous discussed[10, 13-15], but in brief, immune cells will be stimulated with non-specific (LPS, mitogen, BCG) and COVID19-specific antigens with immune function measured by ELISA and flow cytometry. Epigenetic studies are discussed in more below.

## Coordination among participating sites

This is a multi-site study with Houston (BCM, MD Anderson and TAMU) and Los Angeles (Cedars Sinai Medical Center) starting enrollment. Other sites may participate in the United States, but will obtain their own local IRB approval to do so along with executing data use agreements.

All sites will use the same electronic data capture form (REDCap), the same data dictionaries and the same data management plans. Each institution will use independent REDCap “instances” and de-identified data will monthly be exported and be analyzed by the DSMB.

# TREATMENT OF SUBJECTS

## Investigational product/treatment

In the United States, only the FDA approved TICE® BCG (for intravesical use) BCG LIVE strain of the BCG (Merck) vaccine or a saline placebo will be administered. This will be given intradermally (0.1mL) in the deltoid area. We are NOT using the BCG Vaccine USP which is also the Tice strain of BCG as it is indicated for the prevention of *Mycobacterium bovis*, at a different dose, following a different preparation and by means of a different route of administration.

In Houston, randomization will also occur 1:1. (Block randomization discussed below) Placebo vaccine will be 0.1 mL 0.9% NaCl, which is the same amount and color as the intervention.

We remain open to guidance from the FDA and IRBs regarding utilizing additional strains of BCG (eg Russian Strain (Verity), Tokyo Strain (JBL), as they might become available and as necessary). However, we confirm that no other forms of BCG will be administered without IRB and FDA approval. As of 3 April 2020, only the BCG Tice strain will be used in the United States.

# INVESTIGATIONAL PRODUCT

## Name and description of investigational product(s)

Participants that are randomized in the active arm will receive BCG vaccine, the FDA-approved TICE® BCG (for intravesical use) BCG LIVE strain in the United States.

## Summary of findings from non-clinical studies

BCG has been shown to have the ability to train the innate immune system in an in vitro and in vivo setting. eg. when human monocytes are incubated for 24h in vitro with BCG and after a week are re-challenged with another (part of a) pathogen, the cytokine production is increased, compared to non-BCG trained monocytes.[13] BCG also protects in experimental models of viral infections (e.g. influenza) in murine models. ^13^

## Summary of findings from clinical studies

BCG vaccine has been shown to induce training of the innate immune system in vivo, just as heterologous effects of the adaptive immune system (e.g. increased cytokine production by monocytes and lymphocytes of BCG-vaccinated individuals), which is thought to have a protective effect in countries with a high infectious pressure (e.g. most third world countries).^17,18^ After vaccination with BCG, immune cells isolated from healthy individuals react with an increased production of proinflammatory cytokines after stimulation with both specific (MTB) and non-specific (bacterial, viral, fungal) stimuli. Adolescents vaccinated with BCG have shown a 70% decrease in the incidence of respiratory tract infections compared to placebo^9^. In a small Indonesian trial, consecutive BCG vaccination for 3 months reduced the incidence of acute upper tract respiratory infections by 80% (95%CI=22-95%).[7]

## Summary of known and potential risks and benefits

The potential benefit for subjects randomized to the BCG-arm is that administration of the vaccine could prevention SARS-CoV-2 infection and lower risk on developing severe illness caused by SARS-CoV-2.

Potential risks include only the well-known side effects of both the vaccines. A short summary of the most common side effects includes: discomfort at the injection side, scarring at injection side, fevers, nausea, vomiting. Severe but very uncommon side effects are: neurological symptoms such as injection site abscesses, BCG lymphadenitis, disseminated BCG diseases, osteitis, osteomyelitis, anaphylaxis, formation of keloid/lupoid, suppurative lymphadenitis. A complete list of known side effects can be found in the information product insert.

Subjects that receive the placebo hardly have any potential risks and no benefits. Local hematoma formation can occur at the site of vena puncture.

## e. Route of administration and dosage

The TICE® BCG (for intravesical use) BCG LIVE strain will be administered in the upper arm slowly (deltoid area), in about 10 seconds, intradermally 0.1ml of the suspended vaccine, which accounts for approximately 0.075mg of attenuated *Mycobacterium bovis*. The administered 0.1 mL of reconstituted TICE® BCG (for intravesical use) BCG LIVE strain vaccine is approximately 2x10^5^ CFU.

Placebo: Administer at the left upper arm slowly, in about 10 seconds, intradermally 0.1ml of 0.9% NaCl solution.

## g. Preparation and labelling of Investigational Product (IP)

Preparation and labelling of the investigational products are performed

according to GMP guidelines.

In the United States, TICE® BCG (for intravesical use) BCG LIVE strain will be purchased from Merck and reconstituted and prepared according to the package insert/per the approved prescribing information and will include:

a) preparation using aseptic technique during reconstitution as stipulated in the prescribing information for TICE BCG (for intravesical use) BCG Live.

b) that reconstituted TICE® BCG should be kept refrigerated (2–8°C), protected from exposure to direct sunlight, and used within 2 hours of reconstitution.

c) Unused solution should be discarded after 2 hours.

d) Intact vials (prior to reconstitution) of TICE BCG should be stored refrigerated, at 2–8°C (36–46°F).

Merck sells and distributes 1x10^8^ CFU of TICE® BCG (for intravesical use) BCG LIVE strain in a lyophilized (freeze-dried) powder and this will be reconstituted in 50 mL of preservative-free saline, yielding 2x10^6^ CFU/ mL. Administration of 0.1 mL will contain ~2x10^5^ CFU, similar to the quantity of BCG vaccine administered worldwide.

The TICE® BCG (for intravesical use) BCG LIVE strain format of the BCG vaccine available in the United States comes in quantities of 1x10^8^ CFU per vial and must be used within 2 hours of reconstitution).

## h. Drug accountability

Drug accountability will be done in compliance with each pharmacy per their local standard operating procedures and institutional requirements and dispensed from each local pharmacy’s supply.

# STUDY PARAMETERS/ENDPOINTS

## Primary study endpoint

Incidence of rt-PCR-confirmed SARS-CoV2 infection following BCG vaccination compared to that following placebo.

## Secondary study endpoint

In individuals who test positive for Covid-19, the proportion with severe disease following BCG vaccination compared to placebo, as defined by the following necessary care levels: non-hospital care; patient hospitalized but no oxygen required; hospitalized and oxygen required; patient treated in intensive care and/or on mechanical ventilation; patient died.

# STUDY PROCEDURES

## Recruitment, Randomization, treatment allocation, and blinding

A standardized, IRB approved email will be sent to department chairs describing the study. A research coordinator will reach out to interested participants via phone with the help of an IRB-approved verbal script to introduce the study, confirm eligibility and provide further instructions on how to access and sign the IRB-approved ICD via REDCap using their own electronic devices. It is important that we obtain the consent via REDCap to a) avoid direct person-to-person contact and comply with social distancing imposed recommendations, and b) minimize the waste of reconstituted BCG by allowing the research personnel to schedule vaccinations in a controlled fashion.

A person under investigation for COVID is not eligible, as per eligibility criteria. To further prevent risk of nosocomial transmission in case of asymptomatic shedding, consenting and screening will occur electronically and over telephone and the intervention will occur at a designated research location away from patient care. If a study participant develops symptoms of COVID19 after enrollment in the study, they will be tested for COVID19 like any other health care worker or community member, and under the supervision, provisions and regulations of their institution and followed per protocol.

Patient registration into the trial will happen immediately after consent has been provided and will involve entering of baseline information into an electronic data capture system (RedCap).

Once the eligibility is confirmed and the ICD signed by the participant and stored in REDCap, the research coordinator will randomize the participant and communicate the treatment assignment to the nurse administering the vaccination. The nurse will subsequently assign an appointment and communicate date and time of vaccination with the participant. All eligible participants will receive intradermal injections of BCG:placebo in a 1:1 ratio.

Both participants and investigators will be blinded to the treatment assignments during the study. However, in case of an emergency where it is important to know the treatment received, the investigator and/or participant can reach out to the unblinded study personnel who will provide the unblinded data. All participants will receive their treatment allocation at the end of the study, after the data analysis is finalized.

Unblinded personnel will not be involved in the collection and analysis of study data other than the baseline eligibility criteria.

The end of the study is defined as the last patient’s last entry in the electronic data capture system.

Informed Consent and Eligibility

The following types of procedures will be conducted as indicated below:

Medical **history** will be obtained from patient medical record/clinical chart. Informed Consent will be obtained to access these records. When information cannot be obtained or is not available from the patient medical record/clinical chart, it will be obtained via patient interview.

Visual **examination** will be conducted solely to look for existing BCG vaccination scars.

Symptom **evaluation** will be conducted via an electronic survey administered to participants every 7 days.

Height, weight, HIV status **and pregnancy** status will be collected as self-reported information. If unknown, a urine pregnancy test will be performed.

Nasopharyngeal, oral and/ or rectal **swabs** will be collected for rt-PCR test for SARS-CoV2 infection if a participant develops symptoms consistent with Covid-19.

**If a participant does not know their PPD/IGRA status from within the last 24 months (all health care providers should have this information), an IGRA can be performed to evaluate eligibility.**

**Study participants have the option of donating blood** via phlebotomy (for serological test for Covid-19 disease and PBMCs for immune correlates) or providing a fingerstick and dried blood spot (for serologic test for Covid-19).

Data will be collected at four time points/periods: (1) after consent, (2) at baseline, (3) during follow-up period, and (4) at study end.

Data to be collected during screening includes medical history, visual exam results, results of rt-PCR and serological test results.

Data to be collected during baseline enrollment includes eligibility confirmation, demographic information, risk factors, randomization assignment, confirmation of BCG vaccination/placebo, any immediate reactions to BCG vaccination/placebo. Subjects may also be asked to share a picture of just the injection site. Sharing the picture of the injection site is optional and will not be available to blinded staff members until the end of the study.

Data to be collected during follow-up includes intermittent surveys about the presence of flu-like symptoms, rt-PCR test results if done, serological test results, if testing positive for Covid-19 information regarding their disease course, and disease outcome status.

**The following procedures will be performed and/or data collected as listed below data after informed consent is obtained:**

| **Data Variable** |  |
| --- | --- |
| Date of signed Informed Consent Form | X |
| Role in hospital | X |
| Department in hospital | X |
| rt-PCR test for SARS-CoV2 result | X |
| Serological test for Covid-19 result | X |
| Number of BCG scars (by visual examination) | X |
| Medical history* | X |
| Previous PPD and IGRA test results | X |
| History of TB disease | X |
| History of previous HIV testing | X |
| Urine Pregnancy test result (if applicable) | X |
| Plans of pregnancy in 30 days | X |
| Plan to stop working in 3 months/ leave facility in 6 months | X |
| Current diabetes mellitus | X |
| Current chronic kidney disease | X |
| Currently taking immunosuppressive drugs | X |
| Living with someone with HIV, immunocompromised, taking immunosuppressive drug | X |
| Chemotherapy in past 3 months | X |
| History of organ/bone marrow transplant | X |
| Access to smartphone | X |

## Baseline data collection/procedures

The following procedures will be conducted and data collected as indicated below:

A questionnaire to obtain information about age, sex, demographic information, who they live with, smoking status, any current medications they are on, and other comorbidities

Participants will then be randomized to either receive a single dose of BCG vaccination or placebo.

BCG vaccination or placebo will be administered.

Eligibility screening data will carry forward into the trial.

The following additional data points will be collected:

| **Data Variable** |  |
| --- | --- |
| Age | X |
| Sex | X |
| Race | X |
| Ethnicity | X |
| Nationality | X |
| Who they live with | X |
| Height | X |
| Weight | X |
| Smoking status/tobacco use | X |
| Alcohol use | X |
| Current list of medications | X |
| Current list of comorbidities | X |
| History of diabetes mellitus | X |
| History of hypertension | X |
| History of stroke | X |
| History of kidney disease | X |
| History of COPD | X |
| Randomization assignment | X |
| BCG/placebo administered | X |

## Follow-up procedures and data collection:

Participants will be followed to assess whether they get infected with SARS-CoV2:

Participants will complete intermittent surveys via an electronic system every 7 days to assess the presence of any flu-like symptom, including sore throat, fever, headache, malaise, and cough. Note that this is part of routine surveillance for Covid-19 in health workers at the United States site. Consent forms will ask for consent to access this survey information.

Any positive response on the survey will trigger a nasopharyngeal, oral and/ or rectal swab to be collected to test for Covid-19 via rt-PCR

All participants, regardless of survey responses, will have serology for Covid-19 tested at 4 week intervals during the follow-up period (6 months)

If a participant completes the follow-up period and does not test positive for disease, their study participation is complete

If a participant does test positive for disease, their disease status will be ascertained for up to two months or until an outcome is available through one of the following mechanisms: (1) an electronic survey if they are not admitted to the hospital, including questions about the number of days they are ill, daily fever, and other symptoms; or (2) (2) if they are admitted to the hospital, ordinal outcomes for disease severity will be extracted from the hospital’s electronic medical records system.

During the first week of follow-up, all participants will actively be asked about any adverse events; thereafter, participants will report unsolicited AEs through the electronic survey. Vaccine related adverse events will be graded using the FDA guidance (<https://www.fda.gov/media/73679/download>) and noted using WHO-recommended Adverse event following Immunization forms (AEFI; <https://vaccine-safety-training.org/classification-of-aefis.html>).

Participants will have the option of donating 12 mL of blood for plasma (serology) and PBMCs for secondary analysis of immune correlates and for future analysis based on covid19-specific IgM and IgG. If they do not donate 12mL of blood, a fingerstick will be required for baseline COVID19 serology.

Dried Blood Spot (DBS): all participants will self-collect DBS samples at week 4, 8, 12, 16, 20 and 24. Envelopes to store the DBS are provided upon enrollment and can be mailed or dropped off at work and picked up by study coordinators.

COVID specific RNA is found in stool for ~21 days when an individual develops infection (<https://doi.org/10.1038/s41586-020-2196-x>). Participants will have the option of collecting stool swabs monthly if they are asymptomatic or weekly if they develop symptoms. Nucleic acid testing will be performed in retrospect to support secondary objectives.

If participants develop symptoms consistent with COVD19, will be PCR tested for COVID19. They will be given the option of donating 12 mL of blood for plasma and PBMCs 2 weeks after symptoms resolve.

Week 12 (+/- 2 Weeks), participants will be given the option to donate 12 mL of blood for plasma and PBMCs for secondary analysis of immune correlates and for future secondary analysis based on covid-specific IgM and IgG.

Week 24 (+/- 2 Weeks), participants will be given the option to donate 12 mL of blood for plasma and

PBMCs for secondary analysis of immune correlates and for future secondary analysis based on covid-specific IgM and IgG.

Except for the administration of BCG vaccine or placebo and the above mentioned DBS and phlebotomy, participants will undergo no invasive procedures for study purposes.

The following data points will be collected during the follow-up period and at end of study:

| **Data Variable** |  |
| --- | --- |
| Sore throat (collected at multiple time points) | X* |
| Fever (collected at multiple time points) | X* |
| Headache (collected at multiple time points) | X* |
| Malaise (collected at multiple time points) | X* |
| Cough (collected at multiple time points) | X* |
| rt-PCR test for SARS-CoV2 result (as indicated) | X |
| Serological test for Covid-19 result (every 2 weeks) | X |
| Number of days ill | X |
| Daily fever | X |
| Other Covid-19 symptoms | X |
| Admitted to hospital | X |
| Required oxygen | X |
| Treated in intensive care | X |
| Required ventilation | X |
| Death | X |
| Severe pneumonia | X |
| Respiratory failure | X |
| Acute respiratory distress syndrome | X |
| Sepsis | X |
| Septic shock | X |

**Already being collected as part of routine surveillance of health care workers. Will request access to this information in Informed Consent Form.*

## Withdrawal of individual subjects/End of Study

Subjects can leave the study at any time for any reason if they wish to do so without any consequences. The investigator can decide to withdraw a subject from the study for urgent medical reasons. Participants who received placebo will be unblinded at the end of the study and pending a recommendation by the DSMB, they will be offered the option of receiving the BCG intervention.

## Replacement of individual subjects after withdrawal

A participant will only be replaced in case of withdrawal before the administration of BCG vaccine/placebo.

## Rationale:

A potential additional benefit of BCG vaccination is the impact of the vaccine on improving cognition and Alzheimer’s Disease (AD) outcomes in the presence and absence of COVID19. There are series of published evidence that BCG can improve cognitive outcomes for AD and Parkinson’s[16, 17]. Furthermore, there is mounting evidence that COVID-19 can impact the CNS[18-21], as evidenced by the loss of smell and taste reported by many patients as well as the presence of dementia in nearly 40% of patients. Prior pandemics have observed spikes in Parkinson’s and other cognitive disorders afterwards[22] and coronaviruses have been associated with Parkinson’s[23]. The cognition sub-study would involve cognition baseline and MRI (brain imaging) and follow up at 6 months and 2 years and would include BCG vaccinated, placebo-controlled, COVID-exposed and non-exposed groups. This study design will provide an extremely sensitive and longitudinal measure of cognitive ability that will allow examination of the impacts of COVID-19 and vaccination on the trajectory of mental status.

## Premature termination of the study

During the study, interim analyses is planned. The safety and futility of the trial will be monitored carefully by the study team and DSMB during these interim analyses. If warranted, the study will be terminated (prematurely). Description of the DSMB and interim analysis is described further below.

# OPTIONAL COGNITIVE SUB-STUDY (Not available at all sites).

## Population:

400 subjects enrolled in main BADAS study.

## Additional Inclusion Criteria:

- Normal or corrected-to-normal vision and hearing (able to see images on computer screen and hear auditory events delivered through the computer speaker).
- >45 years of age.

## d. Additional Exclusion Criteria:

- Significant history of mental illness, drug or alcohol abuse; severe trauma preventing normal use of dominant hand (needed to move the mouse cursor); clinical depression (unless medically controlled); other neurologic conditions (i.e. stroke), or learning disability; ophthalmologic/visual problems that prevent viewing a computer screen at a normal distance (such as legal blindness, detached retinas, occlusive cataracts).
- Having pacemakers, aneurysm clips, cochlear implants, pulse oximeters, EKG leads, or other metal/foreign objects in body or face, making them unable to receive **MRI.**
- <45 years of age.

## Study Endpoints (Additional to Main Study):

Primary endpoint: incidence of COVID-19-related cognitive impairment.

Secondary endpoint: severity of cognitive impairment following BCG vaccination compared to placebo that is COVID-19-related.

## Additional Risks Beyond Main Study:

Minimal risk, MRI and clinical cognitive tests are minimal risk and only involve a small amount of additional time at each visit, expected to be about 2 h in duration, including MRI, with three visits total.

## Clinical Cognitive Assessment (CCA):

A rapid and sensitive assessment of cognitive status will be accomplished using a cognitive battery with multiple forms designed to identify subtle changes in high functioning medical staff. We have chosen assessments that reduce human cost and produce low variability in data, minimizing potential error. A computer-based process will be used to accomplish high sensitivity and low variability in our cognitive assessments. We will use the NIH Toolbox Cognition Batteries that have strong psychometric assessment capabilities, appropriate procedural considerations, the ability to integrate with REDCap and utilize tablet computer for the process. This system will minimize human factor and related costs with unlimited administrations and scoring capability. We will utilize the ability of NIH Toolbox to assess integrated higher level aspects of cognition where cognitive changes are most impactful, including: attention, working memory, executive functioning and processing speed measures. This strategy will allow us to identify subtle changes in cognitive function due to COVID-19 and the ability of BCG vaccination to mitigate them.

Details for NIH Toolbox Cognition Batteries are available online: <http://www.healthmeasures.net/explore-measurement-systems/nih-toolbox/intro-to-nih-toolbox/cognition>

## Magnetic Resonance Imaging (MRI):

Structural imaging by MRI allows sensitive assessment of most cognitive disorders, a partial list of which includes: dementia, Alzheimer’s, Parkinson’s, mild cognitive impairment, vascular cognitive impairment, and cerebrovascular disease. When combined with longitudinal imaging, MRI becomes even more sensitive and can establish cognitive status trajectory well before these neuropathologies are clinically obvious[24-26]. Whole brain high-resolution MRI with coronal slices will be used to image the entire brain, hippocampus and temporal lobe to assess the extent of regional atrophy, including volume loss, and cerebrovascular disease using both visual rating scales and automated segmentation methods to measure cognitive changes. Measures will include assessment of vascular dementia, postero-medial parietal lobe atrophy, posterior cingulate gyrus, hippocampal-mediated temporal lobe, atrophy in inferior frontal lobe, visual cortex, insula, hypothalmus, midbrain, caudate, putamen, anterior hippocampi, macrohaemorrhage, fronto-temporal degeneration, atrophy of medial temporal structures and whole-brain atrophy. This strategy will provide a sensitive measure of cognitive status at each time point and trajectory that will ensure we obtain rapid insight into the effects of COVID-19 and BCG vaccination on cognition.

# SAFETY REPORTING

## Temporary halt for reasons of subject safety

The investigators will suspend the study if there is sufficient ground that continuation of the study will jeopardize subject health or safety. The investigator will notify the IRB without undue delay of a temporary halt including the reason for such an action. The study will be suspended pending a further positive decision by the IRB. The investigator ensure that all subjects are kept informed.

## AEs, SAEs and SUSARs

### Adverse events (AEs)

Adverse events are defined as any undesirable experience occurring to a subject during the study, whether or not considered related to the investigational product, the placebo or the trial procedures. Adverse events can be self-reported by the subjects using the mobile application. All adverse events reported spontaneously by the subject or observed by the investigator or his staff will be recorded and assessed per institutional IRB and policies.

### Expected mild Adverse events

Although BCG vaccination almost always causes local reactions, serious or long-term complications are rare. Almost all recipients of BCG will experience vaccine site erythema and development of a small pustule. Slight tenderness at the puncture site may be encountered as well as some itching. The initial skin lesions usually appear within 10–14 days and consist of small red papules at the site. The papules reach maximum diameter (about 3 mm) after 3 to 4 weeks, after which they may scale and then slowly subside.

Theoretically it is feasible that a HCW BCG vaccinated could shed attenuated live BCG and transmit this to an immunocompromised patient. While theoretically feasible, we found no case reports of such events. Further, the ability to keep the vaccine site covered is an exclusion criterion. Study participants will be notified of the expected pustule formation and the need to keep it covered by gauze and clothes during the period it is draining. Before the pustule drains, it has characteristic fluctuant appearance and should be covered with gauze from the time fluctuance develops.

### Expected rare, serious Adverse events:

Approximately 1 in 1000 to 1 in 10,000 will experience suppurative lymphadenopathy including moderate axillary or cervical lymphadenopathy and induration and subsequent large pustule formation. While local usually resolved within 3-4 weeks, supportive lymphadenopathy can persist for as long as 3-6 months after vaccination. There is not definitive management for suppurative lymphadenitis and treatment will be managed on a case by case basis with some undergoing “watchful surveillance and others drained with incision and drainage. All adverse events will be discussed by the site and overall PIs.

More serious local reactions include ulceration at the vaccination site, regional suppurative lymphadenitis with draining sinuses, and caseous lesions or purulent draining at the puncture site. These manifestations might occur up to 5 months after vaccination and could persist for several weeks. The intensity and duration of the local reaction depends on the depth of penetration of the multiple puncture device and individual variations in patients’ tissue reactions.

The most serious complication of BCG vaccination is disseminated BCG infection. The most frequent disseminated infection is BCG osteomyelitis (0.01 to 43 cases per million doses of vaccine administered) which usually occurs 4 months to 2 years after vaccination. Fatal disseminated BCG infection has occurred at a rate of 0.06–1.56 cases per million doses; these deaths occurred primarily among immunocompromised persons. Disseminated BCG will be treated and managed by each site PI and treated with antibiotics (rifampicin, isoniazid, ethambutol). The risk of anaphylaxis to BCG has been described but is very rare. In a study of over 50,000 individuals who received BCG or placebo, anaphylaxis occurred in no individuals receiving BCG.[27] The vaccination will take place within 0.5 mile of a hospital in the event this occurs. There is nowhere in the TMC area that would not therefore be suitable.

Theoretically, it is feasible that BCG could induce an enhanced innate immune response that induces exuberant immune pathology when an individual is secondarily infected with covid19. Existing human and animal models suggest that BCG will improve antiviral immunity and not induce immunopathology. A recent report in the Lancet suggests that covid related disease is due to suppressed immunity, not due to exuberant immunopathology (Wang To et al <https://doi.org/10.1016/S1473-3099(20)30196-1> Chen and Li, Lancet ID: <https://doi.org/10.1016/S1473-3099(20)30235-8>), but high viral load and lack of host immunity. Participants will be completing daily symptom dairies and results will be reviewed every 4 weeks by a DSMB consisting of immunology, pulmonology, and infectious disease physicians. In personal correspondence from Dr. Lamm (letter available upon request), he reports anecdotal evidence that BCG does not induce an increase in viral infections. Further, in the ACTIVATE trial, which took place this past year, 200 elderly patients in Greece upon hospital discharge received either BCG vaccination or placebo. None have since developed a covid-related ARDS response (letter from Dr. Kanellakopoulou and email from Dr. Netea available upon request.)

### Serious adverse events (SAEs)

An adverse event or suspected adverse reaction is considered “serious” if, in the view of either the investigator or the sponsor, it results in any of the following outcomes:

- Death
- A life-threatening adverse drug experience – any adverse experience that places the patient, in the view of the initial reporter, at immediate risk of death from the adverse experience as it occurred. It does not include an adverse experience that, had it occurred in a more severe form, might have caused death.
- Inpatient hospitalization or prolongation of existing hospitalization
- A persistent or significant incapacity or substantial disruption of the ability to conduct normal life functions.

Important medical events that may not result in death, be life-threatening, or require hospitalization may be considered a serious adverse drug experience when, based upon appropriate medical judgment, they may jeopardize the patient or subject and may require medical or surgical intervention to prevent one of the outcomes listed in this definition. Examples of such medical events include allergic bronchospasm requiring intensive treatment in an emergency room or at home, blood dyscrasias or convulsions that do not result in inpatient hospitalization.

All events occurring during the conduct of a protocol and meeting the definition of a SAE must be reported to the IRB per their policy and institutional guidelines .

### Suspected unexpected serious adverse reactions (SUSARs) / Unanticipated Problems (UP)

- UPs are defined according to each institutional IRB of record. The UP will be defined and reported per institutional policy. All serious adverse events will be reported to local site PI within 24 hours of knowledge of the event.
- Unless stated otherwise in the protocol, all SAEs, expected or unexpected, will be reported to the DSMB as soon as possible, but in all cases within 5 working days of knowledge of the event regardless of the attribution.
- Death or life-threatening events that are unexpected, possibly, probably or definitely related to drug must be reported within 24 hours of knowledge of the event.
- Serious adverse events will be captured from the time of the first protocol-specific intervention, unless the protocol states otherwise, and be reported until 30 days after the last protocol specific data collection timepoint. Serious adverse events must be followed until clinical recovery is complete and standard of care laboratory tests have returned to baseline, progression of the event has stabilized, or there has been acceptable resolution of the event.
- Additionally, any serious adverse events that occur after the 30 day time period, or protocol specific timeline, that are related to the study treatment must be reported to the IRB Office.

## Data Safety Monitoring Board

Safety oversight will be provided by a DSMB. The DSMB consists of 5-7 expert members and are independent of study personnel. The DSMB will consist of members with expertise related to the trial. The DSMB will be run via teleconference with the following current members: Dr. Christina Kao (pulmonologist, BCM), Dr. Daniel Musher (infectious disease physician, BCM), Dr. Naval Daver (oncologist and immunotherapy expert, MD Anderson), Dr. Elizabeth Chiao (infectious disease physician, BCM) and Dr. Edward Graviss, an expert in mycobacterial infections and statistical design and evaluation. If these existing members are unable to participate, they will be replaced with another member with similar expertise. The DSMB will review study collected data and events every month including enrollment, demographics, compliance, adverse events. The DSMB can also meet ad hoc in the event of an unanticipated SAE. The DSMB independent study statistician, Jose Euberto Mendez, will have access to unblinded study data to evaluate these criteria. A final data review meeting will occur two to four months after study completion to review the cumulative safety data. Additional data can be requested by the DSMB as they deem requisite. The DSMB may share de-identified reports with participating/collaborating sites and/or institutions.

## Halting Rules

The DSMB or study PI may halt the study in for the following reasons:

- Two or more participants develop anaphylaxis
- Three or more participants develop disseminated BCG or suppurative lymphadenitis
- Three or more participants develop disseminated BCG-itis
- Any participant experiences a serious unexpected adverse event that could be related to vaccination
- A 5% increase in severe COVID19 related illness (severe pneumonia, respiratory failure, ARDS, sepsis, septic shock) among those vaccinated compared to those that received placebo

## Annual safety report

In addition to the expedited reporting of SUSARs, the Investigator will submit, a summary of safety reports to their IRB, annually, per their local policy and institutional guidelines..

## Follow-up of adverse events

All AEs will be followed until they have abated, or until a stable situation has been reached. Depending on the event, follow up may require additional tests or medical procedures as indicated, and/or referral to the general physician or a medical specialist. SAEs collection and reporting will occur until end of study, as defined in the protocol.

# SAMPLE SIZE AND STATISTICAL ANALYSIS

Data will be reported quantitatively. All analyses will be performed from the intention-to-treat principle. Missing data will be dealt with by multiple imputation using the mice package in R.

## Sample size calculation

The study in the United States will include at least three sites: Houston (BCM and MD Anderson Cancer Center considered as ‘one site’ due to proximity), Texas A&M University (TAMU), and Cedars Sinai Medical Center (CSMC). TAMU will enroll 1400 individuals. The proposed enrollment sample size is designed to provide 80% power to detect a 60% vaccine efficacy (a relative risk of 0.4 among the vaccinated). This is based on the observed three-fold decline in respiratory infections in the South Africa adolescent cohort with 80% power and 0.05 type 1 error in a two-tailed test. We expect 5% of study participants to develop symptomatic COVID19 during follow-up, therefore a sample size of 1400, with 700 receiving intervention and 700 receiving placebo. We do not expect significant loss to follow up among HCWs (there has been zero LTF after the first 500 participants). We will plot Kaplan-Meier curves comparing COVID19 disease between the BCG and placebo groups and report hazard ratios and p-values using Cox proportional hazard models. DBS samples will be collected at baseline and weeks 4, 8, 12, 16, 20 and 24 post intervention. PBMCs and plasma donations are optional at baseline, 12 and 24 weeks. If interim analysis identifies a large proportion of participants to be seropositive before intervention, power analysis will be evaluated and an amendment will be submitted if additional participants need to be recruited.

## Interim analysis

One month after the first 100 participants are enrolled, an interim analysis will be held by the independent statistician of the trial. Data will be exported independently from all sites and then data collated and merged for the interim analysis). The above described statistical analysis will be evaluated as well as safety profile. Enrollment is expected to occur within the first two months of launch. Interim analysis will occur based on study enrollment and three to six interim analysis will occur based on temporal dynamics of enrollment as requested by the DSMB. We will stop the clinical trial early if the p-value of any interim analysis is smaller than the pre-specified type-1 error cutoff. Each interim analyses will be reported to the DSMB.

## Primary study parameter(s)

The primary endpoint, development of COVID19 infection, we will use the Cox proportional-hazards model to calculate hazard ratios for the development of Covid-19. This will be reported as the proportion of individuals receiving the intervention who are PCR-positive or seroconvert.

## Secondary study parameter(s)

Disease severity as discussed above and based on guidelines proposed by the World Health Organization. We will use the Chi square for significance to calculate the risk ratios for the development of severe Covid-19 disease. Disease severity will be based on the level of care required for individuals who test positive for COVID19 as follows: non-hospital-based care; patient hospitalized but no oxygen required; hospitalized and oxygen required; patient treated in intensive care and/or on mechanical ventilation; patient died. Additional WHO criteria for severity include severe pneumonia, respiratory failure, acute respiratory distress syndrome, sepsis and septic shock. Days absent will also be self-reported via the RedCap Research App. Immunology and epigenetic studies for innate training be implemented, but in brief, immune cells will be stimulated with non-specific (LPS, mitogen, BCG) and COVID19-specific antigens with immune function measured by ELISA and flow cytometry. Epigenetic studies are discussed in more detail below.

## Other study parameters

Continuous baseline characteristics will be reported as mean and standard deviation or median and inter-quartile range, as appropriate. Categorical baseline characteristics will be reported as count and percentage. No statistical testing for baseline characteristics will be performed.

## Synthesis

In this trial, the BCG vaccine will be used in hospital personnel during the epidemics to prevent infection with the new SARS-CoV-2 virus. The risk of the BCG vaccine is very well known (see product inserts) and only minor local side effects (redness, local pain) are common. The benefits of BCG vaccination based on earlier clinical studies are expected to be a reduction in infection and morbidity of at least 25% compared to unvaccinated individuals, and potentially much higher. These effects are mediated by the capacity of BCG to induce trained immunity and may represent an important tool for protection against SARS-CoV-2 virus infection until a specific effective vaccine is developed.

## Epigenetic and Immune Corollary Studies

COVID19 serology is currently being developed. When available (FDA approved test availability is rapidly evolving and improving), serology from blood drawn at baseline will be used to exclude individuals with preceding COVID19 infection from analysis.

To evaluate if the metabolic-epigenetic-immune mechanisms of the non-specific benefits of BCG for Covid19 are similar to that as previously described (Arts et al 2017), the collection of 12 mL of blood, in sodium heparin tubes, will be option to the participant. Blood will be processed for plasma (~4mL) and PBMCs. PBMCs will be cryopreserved and saved for future epigenetic (ATAC-seq, ChIP-seq, DNA methyl EPIC) and immune functional studies.

## Flow cytometry based immune profiling

A secondary objective of this project is to elucidate the duration and immune correlate of protection of non-specific epigenetic-mediated immune “innate training” by BCG against Covid19. To do so, we will evaluate transcription regulation (DNA methylation, ATAC-seq, Chromatin immunoprecipitation), gene expression (reverse transcription PCR, RNA-seq) and immune phenotyping experiments (ELISAs) and multi-parameter immune phenotyping. We have developed multiple flow cytometry panels to measure the immune response. DNA methylation EPIC and other sequencing based arrays allow for genome wide evaluation of epigenetic silencing. Genome wide evaluation will be validated with site-specific evaluation using PCR as previously described (DiNardo et al JCI 2020; 32125282). Flow cytometry-based immune phenotyping allows 18 parameters to simultaneously be measured. By first stimulating the PBMCs with viral or mycobacterial peptides, we are able to simultaneously **measure and correlate** the quantity and quality of the viral and/ or mycobacterial induced immune phenotype (CD4, CD8, CD36, CD14, CD23), transcriptional changes (T-bet, GATA-3, Foxp3, Helios) and functional immune response such as cytokine release (IL-2, IL-4, IL-10, TGF-β, TNF and IFN-γ) or effector function (perforin, CD107a). COVID19-specific viral peptide mixes are in development and the PBMCs will be saved for a time when measuring COVID19-specific immunity is feasible.

## Epigenomic DNA methylation

BCG induced non-specific immunity has been linked to chromatin conformation and DNA methylation. Therefore, these additional epigenetic studies will be evaluated as part of secondary analysis. The Illumina Infinium MethylEPIC array tests ~850,000 methylation sites including promoters, enhancers and non-island sites. Briefly, genomic DNA (gDNA) will be isolated (DNeasy protocol) from 1x10^6^ PBMCs with >80% viability. From this quantity of PBMCs, I am able to retrieve >1.5μg DNA, which is sufficient to implement this assay (500ng). Experiments only proceed after strict quality control analyzing nucleic acid quality using a Qubit 3.0 fluorometer and Agilent 4200 Tape Station System. I will use the Zymo EZ DNA Methylation kit to bisulfite treat 500ng of gDNA prior to MethylationEPIC testing. Methylation IDAT files will be imported into R statistical system using the Bioconductor (http://www.bioconductor.org) minfi package. After preprocessing and normalization, the Bioconductor limma package will identify probes with greater than 2 or less than 0.5-fold differential methylation with p-values < 0.05. Statistically significant differentially methylated probes will be visualized in Ingenuity Pathway Analysis. Gene ontology (GO) and Gene Set Enrichment Analysis analysis will be implemented using Molecular Signature Database (MSigDB) using hyper-geometric distribution that accounts for multiple comparisons. To evaluate if cell composition is confounding results and to determine the DNA methylation status of specific cell types (CD4, CD14, CD8, NK, and B cells), I will implement the bioinformatic pipelines EDEC (Epigenomic deconvolution) and MeDeCom (methylation decomposition). Both methods utilize linear algebra and classification techniques to quantify the DNA methylation profiles in several steps: 1) identifying optimal number of informative cell subpopulations 2) identify simultaneously both the estimated percentage of and the DNA methylation profile of each cell subpopulation. The explanatory cell subpopulations are then matched to DNA methylation profiles of known references via Spearman correlation. This approach offers multiple benefits: it enables us to assess DNA methylation changes in a cell type specific manner, but in a cost-effective approach because I will be profiling only the heterogeneous blood cells. Validation of bioinformatic EDEC results will then be carried out in magnetic bead purified cell types as described below.

## ATAC-seq

DNA hyper-methylation inversely correlates with chromatin accessibility, however other means of epigenetic silencing exists (i.e H3K27me3, etc.). Therefore to confirm that specific DNA hyper-methylation marks correlate with decreased chromatin accessibility, we will implement ATAC-seq, epigenome-wide chromatin accessibility evaluation. 1x10^5^ cells of interest (identified after EDEC as described above) undergo cell lysis, Tn5-mediated transposition followed by DNA purification, PCR amplification, DNA quality Bioanalysis (using Agilent and Qubit as described above) and sequencing. Bioconductor packages will be used for preprocessing, normalizing and identification of differential chromatin accessibility defined as greater than 2 or less than 0.5-fold difference with p values < 0.05.

## Benefits and risks assessment, group relatedness

The risk to and burden for the subject of BCG vaccination is estimated to be low, according to two previous trials that have been performed with BCG vaccines.[7, 11, 13] The beneficial effect BCG vaccination for the individualized participant is unknown, although the objective is to prevent severe illness to SARS-CoV-2 infection. Using an adaptive design, the study aims to find a positive effect of BCG-vaccine on a population level, which could be applied quickly in participants allocated to placebo and implemented to hospitals that do not participate in the study.

# STRUCTURED RISK ANALYSIS

1. Potential issues of concern

An extensive description of the mechanisms of action of the BCG vaccine, referring to the available in vitro and in vivo evidence, could be found in the Summary of Product Characteristics and the Product Insert.

a. Level of knowledge about mechanism of action

BCG activates NOD2 receptor pathway, and subsequently induces long-term epigenetic changes in chromatin of myeloid cells.[10, 13] The epigenetic reprogramming leads to increased antimicrobial activity of myeloid cells (cytokine production, phagocytosis, reactive oxygen species release). At the same time, BCG induces potent activation of cellular specific immunity, with strong induction of Th1 responses and IFNg release.

1. Previous exposure of human beings with the test product

In a randomized controlled trial, healthy volunteers were vaccinated with placebo or BCG (Baccillus Calmette Guérin) vaccine. These volunteers were injected 14 days latter a tri-valent influenza A vaccine. Volunteers previous vaccinated by BCG developed significantly greater titers against hemagglutinin A of the influenza A virus whereas their circulating monocytes were more potent for the production of IFNγ.[28]

In another randomized placebo-controlled human challenge study, we found that BCG vaccination induced genome-wide epigenetic reprograming of monocytes and protected against experimental infection with an attenuated yellow fever virus vaccine strain. Epigenetic reprogramming was accompanied by functional changes indicative of trained immunity.

In clinical studies followed by clinical endpoints, adolescents vaccinated with BCG have shown a 70% decrease in the incidence of respiratory tract infections compared to placebo.^9^ Consecutive BCG vaccination for 3 months in elderly individuals reduced the incidence of acute upper tract respiratory infections by 80% (95%CI=22-95%). BCG vaccine in immunocompetent adult people is considered safe, even in latently infected adults with prior infant BCG vaccination. In a randomized controlled trial that compared revaccination with BCG versus placebo, no serious adverse events were observed in the BCG arm. ^9^

1. Can the primary or secondary mechanism be induced in animals and/or in *ex-vivo* human cell material?

No

1. Selectivity of the mechanism to target tissue in animals and/or human beings

The effect of BCG is restricted to immune cells, with no effects exerted in other tissues.

1. Analysis of potential effect

The effect of BCG on the susceptibility to / or severity of infection with SARS-CoV-2 is unknown. Previous clinical studies with BCG vaccination that have shown a reduction of respiratory tract infection between 30 to 70%. We hypothesize that BCG could alter the clinical course of SARS-CoV-2 infection and demonstrate 60% efficacy in preventing infection with SARS-CoV2. As mentioned previously, based on a previous trial, we expect rare serious adverse events.

1. Pharmacokinetic considerations

BCG is injected intradermally in the skin at the superior region of the arm, and it is known to persist at the site of injection for up to 4 weeks.

1. Study population

Health-care workers face an elevated risk of exposure to- and infection of- SARS-CoV-2.3. Of 75,465 laboratory-confirmed cases, 2,055 (2.7%) were reported among health care workers from 476 hospitals across China. The majority of cases (77.8%) were found in the working age (30–69 years).

1. Interaction with other products

No known interactions.

1. Predictability of effect

Multiple studies, among which randomized trials showed favorable *in vitro* and *in vivo* protective effects of BCG vaccination in the response to infections, among which viral infections. The objective of the trial is to evaluate if the same effect is observed when using BCG to improve the clinical course of SARS-CoV-2 infection. The safety profile of BCG vaccines has been studied extensively and no severe hazardous effects are expected.

1. Can effects be managed?

The beneficial effects of BCG will be straight forward to register. The local side-effects are minimal and easy to manage. Management of rare and serious adverse events (suppurative lymphadenitis, disseminated BCG and anaphylaxis) will occur by the study participant’s primary medical provider in conjunction with expert advice from infectious disease physicians part of this study.

##

# REGUALTORY REQUIREMENTS/REPORTING, MONITORING AND PUBLICATION

## Financial Disclosure

No financial disclosures to be made. No other commercial support is expended to conduct this study. Merck manufactures the investigational product used in this study and it (or the placebo) will provided for free to subjects participating in this investigation.

## IRB Approval

Prior to initiating the study, the investigators will obtain written IRB approval to conduct the study. Should changes to the study protocol become necessary, protocol amendments will be submitted in writing to the IRB by the investigator for IRB approval prior to implementation. Annual progress reports will be submitted to the IRB annually.

## Ethical conduct of the study

The Investigator agrees, to adhere to the instructions and procedures described in the protocol and conduct the study in accordance with the Code of Federal Regulations (21 CFR Parts 11, 50, 54, 56, 312, 314, and 320), which originate from the ethical principles laid down in the current revision of the Declaration of Helsinki, GCP, and policies and procedures as outlined by the ethical requirements for IRB review and informed consent forms.

## Ethics and regulatory review

A copy of the protocol, proposed informed consent form, other written subject information, and any proposed advertising material must be submitted to the IRB for written approval. The investigator will notify the IRB of deviations from the protocol or serious adverse events/unanticipated problems occurring in accordance with local procedures.

The investigator is responsible for obtaining annual IRB approval /renewal throughout the duration of the study. The IRB will be notified of completion or termination of this study and sent a copy of the study synopsis in accordance with applicable timelines.

## Informed consent

The investigator, sub-investigators, and/or designated staff will explain all aspects of the study in lay language and answer all of the subject’s questions regarding the study. If the subject desires to participate in the study, s/he will be asked to sign the Informed Consent. No study procedure will be performed prior to signing Informed Consent. All study subjects will be given a copy of the signed Informed Consent(s). Prior to performing any study-related activities under this protocol, written informed consent with the approved ICF must be obtained from the subject or subject’s legally authorized representative, as applicable, in accordance with local practice and regulations. The background of the proposed study, procedures, benefits and risks and voluntariness of this study will be explained to the subject.. Full confidentiality of subjects and subject records will be provided according to institutional guidelines. All study subjects will be given a copy of the Informed Consent(s) and given sufficient time to consider whether to participate in the study. The signed consent form will be retained with the study records.

As this study recruits employees, the ICF will also clearly state that involvement is voluntary and not include any coercive language. It will clearly be stated that there will be no favoritism, consternation, retribution or compelling socioeconomic benefits with the participation or refusal to participate in this study.

## Incentives and Research Related Injury

Patients will not be compensated for participating in this study. Participants will not be charged for the cost of the vaccination (or placebo). If a subject are injured, necessary facilities, emergency treatment and professional services will be available to them, just as they are to the general community.

In the event of injury resulting from this research, or negligence of local study personnel, the study team nor their institution are able to offer financial compensation nor to absorb the costs of medical treatment.

Procedures and treatment for clinical care related to potential adverse events will be billed to the subject and/or their insurance or applicable third party.

## Changes to the Protocol and/or Informed Consent

Changes to the research covered by this protocol must be implemented by formal protocol amendment. Protocol amendments must not be implemented without prior IRB approval. When the change(s) involve only logistic or administrative aspects of the study, the IRB only needs to be notified. The data recorded on the CRF and source documents will reflect any departure from the protocol and the source documents will describe the departure and the circumstances requiring it.

A ‘substantial amendment’ is defined as an amendment to the terms of the IRB application, or to the protocol or any other supporting documentation, that is likely to affect to a significant degree:

- the safety or physical or mental integrity of the subjects of the trial;
- the scientific value of the trial;
- the conduct or management of the trial; or
- the quality or safety of any intervention used in the trial.

## Annual progress report

The sponsor-investigator will submit a summary of the progress of the trial to their IRB once a year. Information will be provided on the date of inclusion of the first subject, numbers of subjects included and numbers of subjects that have completed the trial, serious adverse events/ serious adverse reactions, other problems, and amendments.

## Training of site study personnel

The Investigator will assure research activities, including those study-related duties delegated and will be performed by appropriately qualified individuals. The Investigator will assure that study staff will demonstrate due diligence in recruiting and screening study subjects.

The investigator will maintain a list of appropriately qualified persons to whom he/she has delegated study duties. All persons authorized to make entries and/or corrections on case report forms will be included on the Delegation of Authority Form. The investigator and study staff will be responsible for maintaining a comprehensive and centralized filing system of all study-related (essential) documentation, suitable for inspection at any time by applicable regulatory authorities, including completed case report forms, subject identification list, study files and all correspondence to and from the IRBs.

## Study Management

Each site Investigator will assure proper implementation and conduct of the study will be performed according to the currently approved study protocol.

## Data Management, Handling, Storage and Security of data and documents

Data will be handled confidentially. A subject identification code list based will be used to link the data to the subject. The key to the code will be safeguarded by each site in a designated location. The electronic CRF (eCRF) are completed on an ongoing basis during the study. The eCRF will be managed via a secure and confidential system (RedCap).

The site principal investigator is responsible for maintaining accurate, complete and up-to-date records for each subject. The site principal investigator is also responsible for maintaining any source documentation.

The investigator is responsible for complying with the requirements for all assessments and data collection as stipulated in the protocol for each subject in the study. For subjects who withdraw prior to completion of all protocol-required data collection and are unable or unwilling to complete, the investigator or study staff can search publically available records [where permitted]) to ascertain survival status. This ensures that the data set(s) produced as an outcome of the study is/are as comprehensive as possible.

The investigator and master file and the electronic data from the eCRF will be stored for a duration of 7 years. All information, data, and results that originate from this study may not be disclosed without the written permission of the principal investigator.

## Temporary halt and end of study report

The sponsor will notify the IRB immediately of a temporary halt of the study, including the reason of such an action. In case the study is ended prematurely, the sponsor will notify the IRB within 15 days, including the reasons for the premature termination. Within one year after the end of the study, the investigator/sponsor will submit a final study report with the results of the study, including any publications/abstracts of the study, to the the IRB. The sponsor will notify the IRB of the end of the study within a period of 90 days. The end of the study is defined as the last patient’s last entry into the application. The sponsor will notify the IRB immediately of a temporary halt of the study, including the reason of such an action. In case the study is ended prematurely, the sponsor will notify the IRB and the competent authority within 15 days, including the reasons for the premature termination. Within one year after the end of the study, the investigator/sponsor will submit a final study report with the results of the study, including any publications/abstracts of the study, to the IRB.

## Monitoring and Quality Assurance

We classify this trial as a low risk study, with a negligible risk because the vaccine is already FDA-approved, and has been shown to be safe in over 3 billion previously administered doses. It has a good safety profile.

## Audits and Inspections

The Investigator will assure that study staff cooperate with monitoring and audits. The Investigator agrees to allow auditing of all essential clinical study documents by the FDA or other appropriate regulatory authorities. Auditing visits will be scheduled with the appropriate staff at mutually agreeable times as applicable.

## Confidentiality and Reporting of Results

To maintain subject confidentiality, all data submitted for the current study will be coded using numeric identifiers only. Only on-site research staff will have access to records that may identify subjects. Any paper research and clinical records will be stored on site in a locked cabinet in a secure location. Electronic records will be accessible only by data management staff, clinical monitors and active site personnel who have furnished the required training and credentials. Subject information will not be released without written permission, except as necessary for monitoring by the FDA.

By participating in this protocol, the Investigator agrees that within local regulatory restrictions and ethical considerations or any regulatory agency may consult and/or copy study documents in order to verify data.

## Public disclosure and publication policy

The results of this study will be disclosed unreservedly at the end of the study. Results that are important for public health will be notified to the competent authorities as soon as possible (RIVM, WMO). The trial will be registered in a public trial registry before the first patient is consented. A description of this clinical trial will be available on <http://www.ClinicalTrials.gov> , as required by U.S. Law. This Web site will not include information that can identify subjects. At most, the Web site will include a summary of the results. Prospective and current subjects can search this Web site at any time.

REFERENCES

1. Benn CS, Fisker AB, Rieckmann A, Sorup S, Aaby P: **Vaccinology: time to change the paradigm?** *The Lancet Infectious diseases* 2020.

2. Hegarty P KA, Zafirakis H, DiNardo A: **BCG vaccination may be protective against Covid-19**. *Researchgate* 2020.

3. Huang C, Wang Y, Li X, Ren L, Zhao J, Hu Y, Zhang L, Fan G, Xu J, Gu X *et al*: **Clinical features of patients infected with 2019 novel coronavirus in Wuhan, China**. *Lancet* 2020, **395**(10223):497-506.

4. Zhang J, Zhou L, Yang Y, Peng W, Wang W, Chen X: **Therapeutic and triage strategies for 2019 novel coronavirus disease in fever clinics**. *The Lancet Respiratory medicine* 2020, **8**(3):e11-e12.

5. Benn CS, Netea MG, Selin LK, Aaby P: **A small jab - a big effect: nonspecific immunomodulation by vaccines**. *Trends Immunol* 2013, **34**(9):431-439.

6. Nemes E, Geldenhuys H, Rozot V, Rutkowski KT, Ratangee F, Bilek N, Mabwe S, Makhethe L, Erasmus M, Toefy A *et al*: **Prevention of M. tuberculosis Infection with H4:IC31 Vaccine or BCG Revaccination**. *The New England journal of medicine* 2018, **379**(2):138-149.

7. Wardhana, Datau EA, Sultana A, Mandang VV, Jim E: **The efficacy of Bacillus Calmette-Guerin vaccinations for the prevention of acute upper respiratory tract infection in the elderly**. *Acta Med Indones* 2011, **43**(3):185-190.

8. Han RF, Pan JG: **Can intravesical bacillus Calmette-Guerin reduce recurrence in patients with superficial bladder cancer? A meta-analysis of randomized trials**. *Urology* 2006, **67**(6):1216-1223.

9. Netea MG: **Training innate immunity: the changing concept of immunological memory in innate host defence**. *European journal of clinical investigation* 2013, **43**(8):881-884.

10. Arts RJW, Moorlag S, Novakovic B, Li Y, Wang SY, Oosting M, Kumar V, Xavier RJ, Wijmenga C, Joosten LAB *et al*: **BCG Vaccination Protects against Experimental Viral Infection in Humans through the Induction of Cytokines Associated with Trained Immunity**. *Cell Host Microbe* 2018, **23**(1):89-100 e105.

11. Hatherill M, Geldenhuys H, Pienaar B, Suliman S, Chheng P, Debanne SM, Hoft DF, Boom WH, Hanekom WA, Johnson JL: **Safety and reactogenicity of BCG revaccination with isoniazid pretreatment in TST positive adults**. *Vaccine* 2014, **32**(31):3982-3988.

12. Hegarty PK, Sfakianos JP, Giannarini G, DiNardo AR, Kamat AM: **COVID-19 and Bacillus Calmette-Guerin: What is the Link?** *Eur Urol Oncol* 2020, **3**(3):259-261.

13. Kleinnijenhuis J, Quintin J, Preijers F, Joosten LA, Ifrim DC, Saeed S, Jacobs C, van Loenhout J, de Jong D, Stunnenberg HG *et al*: **Bacille Calmette-Guerin induces NOD2-dependent nonspecific protection from reinfection via epigenetic reprogramming of monocytes**. *Proceedings of the National Academy of Sciences of the United States of America* 2012, **109**(43):17537-17542.

14. Arts RJW, Carvalho A, La Rocca C, Palma C, Rodrigues F, Silvestre R, Kleinnijenhuis J, Lachmandas E, Goncalves LG, Belinha A *et al*: **Immunometabolic Pathways in BCG-Induced Trained Immunity**. *Cell Rep* 2016, **17**(10):2562-2571.

15. DiNardo A, Rajapakshe K, Nishiguchi T, Mtetwa G, Grimm SL, Dlamini Q, Kahari J, Mahapatra S, Kay AW, Maphalala G *et al*: **DNA hyper-methylation during Tuberculosis dampens host immune responsiveness**. *The Journal of clinical investigation* 2020.

16. Gofrit ON, Klein BY, Cohen IR, Ben-Hur T, Greenblatt CL, Bercovier H: **Bacillus Calmette-Guerin (BCG) therapy lowers the incidence of Alzheimer's disease in bladder cancer patients**. *PLoS One* 2019, **14**(11):e0224433.

17. Faustman DL: **Benefits of BCG-induced metabolic switch from oxidative phosphorylation to aerobic glycolysis in autoimmune and nervous system diseases**. *J Intern Med* 2020.

18. Nath A: **Neurologic complications of coronavirus infections**. *Neurology* 2020.

19. Yuki K, Fujiogi M, Koutsogiannaki S: **COVID-19 pathophysiology: A review**. *Clinical immunology* 2020, **215**:108427.

20. De Felice FG, Tovar-Moll F, Moll J, Munoz DP, Ferreira ST: **Severe Acute Respiratory Syndrome Coronavirus 2 (SARS-CoV-2) and the Central Nervous System**. *Trends Neurosci* 2020.

21. Mao L, Jin H, Wang M, Hu Y, Chen S, He Q, Chang J, Hong C, Zhou Y, Wang D *et al*: **Neurologic Manifestations of Hospitalized Patients With Coronavirus Disease 2019 in Wuhan, China**. *JAMA Neurol* 2020.

22. Henry J, Smeyne RJ, Jang H, Miller B, Okun MS: **Parkinsonism and neurological manifestations of influenza throughout the 20th and 21st centuries**. *Parkinsonism Relat Disord* 2010, **16**(9):566-571.

23. Fazzini E, Fleming J, Fahn S: **Cerebrospinal fluid antibodies to coronavirus in patients with Parkinson's disease**. *Mov Disord* 1992, **7**(2):153-158.

24. Narayanan L, Murray AD: **What can imaging tell us about cognitive impairment and dementia?** *World J Radiol* 2016, **8**(3):240-254.

25. Duchesne S, Bocti C, De Sousa K, Frisoni GB, Chertkow H, Collins DL: **Amnestic MCI future clinical status prediction using baseline MRI features**. *Neurobiol Aging* 2010, **31**(9):1606-1617.

26. Frisoni GB, Fox NC, Jack CR, Jr., Scheltens P, Thompson PM: **The clinical use of structural MRI in Alzheimer disease**. *Nat Rev Neurol* 2010, **6**(2):67-77.

27. **Randomised controlled trial of single BCG, repeated BCG, or combined BCG and killed Mycobacterium leprae vaccine for prevention of leprosy and tuberculosis in Malawi. Karonga Prevention Trial Group**. *Lancet* 1996, **348**(9019):17-24.

28. Leentjens J, Kox M, Stokman R, Gerretsen J, Diavatopoulos DA, van Crevel R, Rimmelzwaan GF, Pickkers P, Netea MG: **BCG Vaccination Enhances the Immunogenicity of Subsequent Influenza Vaccination in Healthy Volunteers: A Randomized, Placebo-Controlled Pilot Study**. *The Journal of infectious diseases* 2015, **212**(12):1930-1938.
