# Supplementary material for: Bacillus Calmette-Guérin vaccination as defense against SARS-CoV-2 (BADAS): a randomized controlled trial to protect healthcare workers in the USA by enhanced trained immune responses
Source: Trials. 2023 Oct 4;24:636. doi: 10.1186/s13063-023-07662-w (PMC10548680; doi:10.1186/s13063-023-07662-w)
Supplement: Supplementary file 2 — Additional file 2. [file 13063_2023_7662_MOESM2_ESM.pdf]

Consent

Supplementary Information
Supplement 2

Please complete the survey below.

Thank you!

Please download the informed consent form:

[Attachment: "TAMU_ICF.pdf"]

If you have any questions about the study or the consent form, please contact the study team at the numbers listed in the consent form or at BADASTrial@lists.tamu.edu

Did you download and read the informed consent document?	◯ Yes ◯ No
Did you understand all the information listed in the informed consent?	◯ Yes ◯ No<br>(if not, please reach out to the study team at the numbers provided so they can answer any questions)
Do you understand that if you agree to take part in this study you have an equal probability of receiving the BCG vaccine or a placebo injection? And that you won't be told what group you are in until after the study ends?	◯ Yes ◯ No
I hereby agree to participate in the research study described to me during the informed consent process and described in this informed consent form.	◯ Yes ◯ No

NOTE: The following cognitive sub-study is only available at the Round Rock/Austin and Temple study sites.

I hereby agree to participate in the optional cognitive sub-study described to me during the informed consent process and described in this informed consent form.	◯ Yes<br>◯ No

NOTE: You may choose to provide biological samples from the following optional study procedures at any time point (excluding the optional cognitive sub-study).

I hereby agree to provide whole blood for immune testing and future COVID-19-related research as described to me during the informed consent process and described in this informed consent form.	◯ Yes<br>◯ No
I hereby agree to provide rectal swabs for future COVID-19-related research during the follow-up period as described to me during the informed consent process and described in this informed consent form.	◯ Yes<br>◯ No
I hereby agree that my identifiable health information may be used and/or disclosed in accordance with this "Informed Consent" document.	◯ Yes ◯ No

First Name

Last Name

Signature

Date & Time

INFORMED CONSENT

INFORMED CONSENT/AUTHORIZATION FOR PARTICIPATION IN RESEARCH WITH OPTIONAL PROCEDURES

**Study Title:** Bacillus Calmette-Guérin (BCG) Vaccination as Defense Against SARS-CoV-2. A Randomized Placebo-Controlled Trial to Protect Health Care Workers by Enhanced Trained Immune Responses	
**Subtitle:** BADAS	**TAMU Study ID:** IRB2020-0432
**TAMU Principal Investigator**: Jeffrey D. Cirillo, Ph.D.	
**TAMU Clinical Investigators:** Gabriel A. Neal, M.D.; George Udeani, PharmD, DSc	
**Additional TAMU Investigator-Wearable Devices Sub-study:** Roozbeh Jafari, Ph.D.	
**Additional TAMU Investigator-Cognitive Sub-study:** Jessica Benard, Ph.D.	
**Funding:** This study is supported by funds from Texas A&M University; The wearable devices sub-study is supported by funds from Philips Research North America	

This is an informed consent and authorization form for a research study. It includes a summary about the study. A more detailed description of procedures and risks is provided after the summary.

STUDY SUMMARY

The goal of this clinical research study is to learn if a Bacillus Calmette-Guérin (BCG) vaccination can help to prevent SARS-CoV2 (COVID-19) infection and/or lessen the severity of the illness amongst high-risk individuals compared to placebo.

High risk individuals include:

- Health Care Workers - Personnel working in a healthcare setting, at a hospital, medical center or clinic (veterinary, dental, ophthalmology).
- First responders - paramedics, firefighters, or law enforcement.
- Those at high risk for severe disease including the elderly and those with comorbidities (having more than one illness or disease at the same time) including obesity, hypertension, diabetes, smokers and reactive airway disease (asthma, COPD).
- Individuals at increased risk of infection because of their decreased ability to limit exposure including racial and ethnic minorities, teachers, police, restaurant wait-staff, delivery personnel, grocery store and retail workers.

A placebo is not a drug. It looks like the study drug but is not designed to treat any disease or illness. It is designed to be compared with a study drug to learn if the study drug has any real effect. In this study the placebo is a saline solution.

This is an investigational study. BCG is FDA approved for treating bladder cancer in the U.S. and has been used throughout the world for vaccination against other diseases like tuberculosis. The researchers are testing a new use for the BCG vaccine to see if it can prevent infection with COVID-19.

It is possible that BCG may help to prevent infection with COVID-19 or lessen the severity of the illness if it is caught. Future patients may benefit from what is learned. There may be no benefits for you in this study.

Your participation is completely voluntary. Before choosing to take part in this study, you should ask questions and discuss with the study team any concerns you may have, including side effects, potential expenses, and the amount of time it will take to participate.

You can read a full list of likely side effects below in the Possible Risks section of this consent.

You will receive the vaccine or the placebo one time.

Your active participation on this study will last about 6 months, though it may be longer if you contract COVID-19 (this is explained later in this document).

The BCG vaccine or placebo will be provided at no cost to you.

You may choose not to take part in this study.

STUDY DETAILS

This is a multicenter study and up to 1400 participants will be enrolled study wide. Up to 700 will take part at Texas A&M University sites.

Randomization

If you agree to take part in this study, you will be randomly assigned (as in a flip of a coin) to receive either the BCG vaccine or placebo. This is done because no one knows if one study group is better, the same, or worse than the other group. You will have a 50% chance (1 in 2) of receiving either vaccine or placebo.

Neither you nor the study researchers will know if you are receiving the vaccine or the placebo. However, if needed for your safety, the study staff will be able to find out what you are receiving.

Study Drug Administration

Before you receive the vaccine or placebo, blood (about 1 teaspoon) will be drawn to test for COVID-19 antibodies. An antibody is a protein found in the blood. You will not have to wait for results before you receive the vaccine or placebo. This sample may be collected as a dried blood spot (DBS) test. This is similar to the finger-stick blood collection that diabetics often use to perform their blood sugar tests.

You will receive the BCG vaccine or placebo as an injection (shot) into the upper arm by trained personnel. The study team will schedule an appointment for you and will tell you when and the location where the injection will be given. The vaccination site will be set-up to minimize close contact and maintain social distancing.

Follow-Up

During the 6 months after you receive the BCG vaccine or placebo, you will receive weekly notifications and be asked if you have any symptoms of COVID-19 (such as sore throat, fever, headache, feelings of discomfort, and cough).

We may contact you if we have questions related to your study participation or if you fail to answer on a timely basis, a study member will call you on the phone to collect missing data. If you cannot be contacted, the study staff may request your medical records to gather this information. This information may include dates of (if applicable) hospital admission, ICU admission, and/or death.

If you do report COVID-19 symptoms, you will be referred to have a swab test to confirm infection.

Additionally, you will collect dried blood spot (DBS) test samples on your own for COVID-19 antibody testing every 4 weeks during the follow-up period. You will be given instructions on how to collect and store the DBS samples, including resealable bags that can be dropped off at designated locations and will be picked up by study staff to minimize contact and time away from duties.

If you complete the 6-month follow-up period and do not test positive for COVID-19, your study participation will be complete unless you agree to the optional follow-up period. If you test positive for COVID-19 at any time, the study staff follow you until the end of the illness.

If the study analysis shows that the BCG vaccine is beneficial, and you were given the placebo, you will be given the option to receive the BCG vaccine. We will contact you and let you know when the vaccine is available.

Optional Follow-Up Period

If you agree, we will extend the follow-up period for an additional 6 months for a total follow-up period of 12 months. During the 6 month optional follow-up period, you will be asked to (1) complete the electronic health survey that examines COVID-19

symptoms on a monthly basis. This is the same survey completed during the regular follow-up period but it is completed once per month instead of weekly; (2) report any COVID-19 related procedures or testing; and (3) report any additional vaccines taken.

If you experience any COVID-19 symptoms or have a positive COVID-19 test during the optional follow-up period we will also ask you to provide 12mL of blood through a phlebotomy procedure (blood draw) approximately one month after the onset of COVID-19 symptoms or the date of a positive COVID-19 test. At the end of the 6 month optional follow-up period, you will be asked to provide an additional 12mL blood sample whether or not you have had COVID-19 symptoms or a positive COVID-19 test during this follow-up period.

REASONABLY FORESEEABLE RISKS

While on this study, you are at risk for side effects. You should discuss these with the study doctor. The more commonly occurring side effects are listed in this form, as are rare but serious side effects. You may also want to ask about uncommon side effects that have been observed in small numbers of patients but are not listed in this form. Many side effects go away shortly after treatment is stopped, but in some cases side effects may be serious, long-lasting or permanent, and may even result in hospitalization and/or death.

Side effects will vary from person to person, and some may occur after you have stopped receiving treatment. Tell the study staff about any side effects you may have, even if you do not think they are related to the study vaccine or study procedures.

BCG Vaccine Side Effects

You should expect skin redness and a blister at the injection site. You may also experience tenderness and/or itching at the injection site. Otherwise, side effects to the vaccine are extremely rare.

In fewer than 1 in 1,000 patients, the vaccine may cause lymph node swelling or inflammation.

In fewer than 1 in 100,000 patients, the vaccine itself can cause an infection known as disseminated BCG infection where it can infect different organs such as the lungs or the bones. Symptoms of infection may include fever, pain, redness, and difficulty breathing.

Blood draws may cause pain, bleeding, and/or bruising. You may faint and/or develop an infection with redness and irritation of the vein at the site where blood is drawn. Frequent blood collection may cause anemia (low red blood cell count), which may create a need for blood transfusions.

Questionnaires may contain questions that are sensitive in nature. You may refuse to answer any question that makes you feel uncomfortable. If you have concerns about completing the questionnaire, you are encouraged to contact your doctor or the study chair.

Researchers will take appropriate steps to keep your information private. However, there is no guarantee of absolute **privacy**.

This study may involve unpredictable risks to the participants.

Pregnancy Related Risks

Taking part in this study can result in risks to an unborn or breastfeeding baby, so you should not become pregnant or breastfeed a baby while on this study. If you can become pregnant, you must use birth control during the 14 days after the dose if you are sexually active.

If you are pregnant, you will not be enrolled on this study. If you become pregnant or suspect that you are pregnant, you must tell your doctor right away.

Getting pregnant will result in your removal from this study.

If you are a female of child-bearing potential and have not been using a reliable method of birth control for at least 30 days, you be given a urine pregnancy test prior to vaccine administration. If the test is positive, you will not be able to participate in the study.

OPTIONAL PROCEDURES FOR THE BCG VACCINE STUDY

Optional Procedure #1: If you agree, blood (about 2 ½ tablespoons) will be drawn for immune testing (a test for past infection) and future COVID-19-related research before you receive the vaccine or placebo and at 12 and 24 weeks after that. If you develop COVID-19 symptoms, an additional sample will be collected approximately 2 weeks after symptoms resolve.

Optional Procedure #2: If you agree, rectal swabs will be collected for future COVID-19-related research during the follow-up period. These will be requested every month if you do not show any symptoms. If you develop COVID-19 symptoms, these will be requested every week. You will be provided with collection kits and instructions by the study staff.

There are no benefits to you for taking part in the optional procedure. Future patients may benefit from what is learned. You may stop taking part at any time. There will be no cost to you for taking part in the optional procedures.

Optional Procedure Risks
Blood draws may cause pain, bleeding, and/or bruising. You may faint and/or develop an infection with redness and irritation of the vein at the site where blood is drawn. Frequent blood collection may cause anemia (low red blood cell count), which may create a need for blood transfusions.

Collecting a **rectal swab** may make you feel uncomfortable.

CONSENT FOR OPTIONAL PROCEDURES

You will be prompted to accept or decline the optional study procedures when you are completing the consent and baseline surveys.

OPTIONAL SUB-STUDY - WEARABLE DEVICES

You must screen into the main BCG vaccine study in order to participate in this sub-study. The decision to participate in this research is voluntary on your part. We plan to enroll up to 200 participants in the wearable devices sub-study.

Sub-Study Details

If you participate in this research, you will be issued a Smart Watch and a Bluetooth ring at no cost to you. Currently, the devices provided for this study are the Garmin Vivoactive 4 Smart Watch and Oura Bluetooth Ring. The information collected from these devices, like your heart rate and temperature ("biometric data"), will be combined with your information from the BCG vaccine study. This information will be processed by our algorithm. Early research indicates that this combination of devices and algorithms *may be* successful at detecting infections. The goal of this study is early detection of symptoms of infection utilizing biometric measurements from a Smart Watch and Smart Ring and the RATE algorithm.

Once you have received these devices, you will need to download the app for each device and set up an Oura and a Garmin International account and sync them with your cell phone. You will need to accept the Garmin and Oura terms of service and privacy policy. If you would like to look at a copy before you sign up, we can provide you with one. Using your cell phone, tablet, or computer you will also be asked to complete the registration process on the Philip's RATE Tracker website at https://www.infectionrisk.philips.com. The investigator will give you a unique participant ID for the registration process that only you and the investigative team will have; that way, your personal information will not be associated with any of your data. You will be asked to provide your email and phone number to enable the investigator to follow up

with you as necessary. After registration, the app will ask a series of demographic questions, like your age and gender. The answers to these questions will help calibrate the Smart Watch and Bluetooth Ring algorithms.

We will ask you to wear to the Smart Watch and Bluetooth Ring as much as possible over the next six months. Additionally, for the duration of the study, you will be asked to open the Garmin app and the Oura app on your phone or tablet at least once every 24 hours, which will automatically sync the data collected by the devices. The devices need to be charged every 3-4 days,

Compensation

There is no compensation for participating in the sub-study. Once your participation has ended, the expectation is that the devices are returned to whomever issued them to you.

Reasonably Foreseeable Risks to your Participation in the Wearable Devices Sub-study

The sub-study will have no direct benefits to you as a participant. There is the potential that the Smart Watch and Bluetooth Ring may cause irritation of your wrists or finger due to the size, weight, and shape of the devices. Additionally, there may be periods of time, due to the unique nature of your work, that the wearing of a Smart Watch and Bluetooth Ring may interfere with your duties or unnecessarily increase your risk of harm or injury. Please follow the standard safety regulations associated with your duties and daily activities, and abstain from wearing the watch and/or ring during activities where they could increase your risk of harm or injury.

The Smart Watch and Bluetooth Ring used in this study are ***not*** medical devices and are ***not*** approved or cleared by the Food and Drug Administration (FDA) for clinically diagnosing any infections or diseases. Your individual results from the wearable devices will not be given to you. If, at any time during this study you have concerns for your health, please contact your primary care provider for advice.

Your biometric data will travel through vendor clouds (Garmin or Oura) using industry standard secure clouds and will arrive at our clouds hosted on amazon web services (AWS) and managed by Philips. Although efforts are made to protect your research study records, there is always a risk that someone could get access to the personal information researchers have stored about you.

OPTIONAL SUB-STUDY - COGNITIVE TESTING

The goal of the sub-study is to learn if the BCG vaccination has any effect on cognition (mental processes) in the presence and absence of COVID-19. There is published evidence that BCG may help improve cognitive outcomes for those with Alzheimer’s disease and Parkinson’s. The researchers want to see if there is any difference between COVID-19 exposed and non-exposed groups. The cognition sub-study would involve cognitive testing and an MRI (brain imaging) before your vaccination, with follow up at 6 months and 2 years.

To qualify for the sub study you must be at least 45 years of age and have normal or corrected-to-normal vision and hearing (able to see images on computer screen and hear auditory events delivered through the computer speaker). You cannot have a significant history of mental illness, drug or alcohol abuse; severe trauma preventing normal use of dominant hand (needed to move the mouse cursor); clinical depression (unless medically controlled); other neurologic conditions (i.e. stroke), or learning disability.

Also, you will not be eligible for the cognitive sub-study if you have any of the following: pacemaker, aneurysm clips, cochlear implants, pulse oximeters, EKG leads or other metal/foreign objects in body or face.

A MRI brain imaging test is considered no more than minimal risk. You will be screened before receiving an MRI. The cognitive tests will involve additional time at each visit, which is expected to be about 2 hours in duration, including MRI. The cognitive sub-study will have three study visits.

Not all study sites will offer the optional sub-study. If you are in an area that offers the sub-study you will be prompted to accept or decline the sub-study when you are completing the baseline surveys for your site.

COSTS AND COMPENSATION

If you suffer injury as a direct result of taking part in this study you should seek medical attention with your usual medical providers. This medical care will be billed to your insurance provider or you in the ordinary manner. You will not be reimbursed for expenses or compensated financially by Texas A&M University for this injury. You may also contact the Texas A&M Institutional Review Board (IRB) with questions about study-related injuries using the information below. By signing this consent form, you are not giving up any of your legal rights.

Samples or data that are collected from you in this study may be used for the development of treatments, devices, new drugs, or patentable procedures that may result in commercial profit.

There are no plans to compensate you for any patents or discoveries that may result from your participation in this research.

You will receive no compensation for taking part in this study.

ADDITIONAL INFORMATION

You may ask the following study team any questions you have about this study:

- Gabriel Neal, MD at (979) 436-0427
- Jeffrey Cirillo, PhD at (979) 436-0343

- George Udeani, PharmD, DSc at 361-221-0700

You may also contact the Texas A&M IRB. The IRB is a committee of doctors, researchers, and community members that is responsible for protecting study participants and making sure all research is safe and ethical. You can contact them at 979-458-4067, toll free at 1-855-795-8636, or by email at irb@tamu.edu. You can talk to them if:

- You cannot reach the research team.
- Your questions, concerns, or complaints are not being answered by the research team.
- You want to talk to someone besides the research team.
- You have questions about your rights as a research participant.
- You want to get information or provide input about this research.

You may choose not to take part in this study without any penalty or loss of benefits to which you are otherwise entitled. You may also withdraw from participation in this study at any time without any penalty or loss of benefits. If you decide you want to stop taking part in the study, it is recommended for your safety that you first talk to the study doctor. Your withdrawal will have no effect on your relationship with Texas A&M University.

This study or your participation in it may be changed or stopped without your consent at any time by the study investigators, the U.S. Food and Drug Administration (FDA), the Office for Human Research Protections (OHRP), or the IRB.

You will be informed of any new findings or information that might affect your willingness to continue taking part in the study, and you may be asked to sign another informed consent and authorization form stating your continued willingness to participate in this study.

Texas A&M University and other participating institutions may benefit from your participation and/or what is learned in this study.

FUTURE RESEARCH

Data

Your data including biometric data may be stored, used or shared with other

researchers for future research studies, and the information gained from your participation in this research study may be published in literature, discussed for educational purposes, and used generally to further science. However, you will not be personally identified when your data and/or information is shared in these ways; all information will be de-identified and aggregated

Samples
Samples (such as blood and/or other biological specimens) are being collected from you as part of this study. Researchers at Texas A&M University or other participating institutions may use any leftover samples that are stored in future research.

Before being used or shared for future research, every effort will be made to remove your identifying information from any data and/or research samples. If all identifying information is removed, you will not be asked for additional permission before future research is performed.

In the case that all of your identifying information is to not be removed before your data or research samples are used for future research, the researchers must get approval from the Institutional Review Board (IRB) before your data and/or research samples can be used. At that time, the IRB will decide whether or not further permission from you is required.

Genetic Research
Samples collected from you as part of this study may be used for genetic research, which may include whole genome sequencing. Whole genome sequencing is a type of testing in which researchers study your entire genetic makeup (DNA). This may help researchers learn how changes in the ordering of genes may affect a disease or response to treatment.

Authorization for Use and Disclosure of Protected Health Information (PHI) or Personally Identifiable Information (PII):

A. During the course of this study, Texas A&M University will be collecting and using your PHI or PII, including identifying information, information from your medical record, and study results. For legal, ethical, research, and safety-related reasons, your doctor and the research team may share your PHI or PII with:
   - Federal agencies that require reporting of clinical study data (such as the FDA, National Institutes of Health [NIH], and OHRP)
   - The IRB and officials of Texas A&M University
   - Collaborating sites: MD Anderson, Baylor College of Medicine, Cedar Siani Medical Center, Philips Research North America, as well as other institutions in the USA and other countries where the vaccine is being studied
   - Study monitors and auditors who verify the accuracy of the information
   - Individuals who put all the study information together in report form

   Study sponsors and/or supporters receive limited amounts of PHI or PII. They may also view additional PHI or PII in study records during the monitoring process. Texas A&M University contracts require sponsors/supporters to protect this information and limit how they may use it.

B. Signing this consent and authorization form is optional but you cannot take part in this study or receive study-related treatment if you do not agree and sign

C. Texas A&M University will keep your PHI or PII confidential when possible (according to state and federal law). However, in some situations, the FDA could be required to reveal the names of participants.

   Once your information is disclosed to parties outside of Texas A&M University, federal privacy laws may no longer protect your PHI or PII.

D. The permission to use your PHI or PII will continue indefinitely unless you withdraw your authorization in writing. Contact the study team to withdraw this authorization at (979) 436-0343 or (979) 436-0427. If you withdraw your authorization, you will be removed from the study and the data collected about you up to that point can be used and included in data analysis. However, no further information about you will be collected.

E. A description of this clinical trial will be available on http://www.ClinicalTrials.gov, as required by U.S. Law. This Web site will not include information that can identify you. At most, the Web site will include a summary of the results. You can search this Web site at any time.

F. There will be no attempt to identify you as a person during the reporting of study results.

CONSENT/AUTHORIZATION

Study Title: Bacillus Calmette-Guérin (BCG) Vaccination As Defense Against SARS-CoV-2. A Randomized Placebo-Controlled Trial To Protect Health Care Workers By Enhanced Trained Immune Responses

If you understand the information in the informed consent and wish to participate in the study, please proceed to the consent survey in RedCap to electronically document your consent.
